# Exploring *Sm*DHODH inhibition: natural products with potential anti-schistosomiasis activity

**DOI:** 10.1007/s40203-026-00628-1

**Published:** 2026-05-08

**Authors:** Rafaela Molina de Angelo, Michell de Oliveira Almeida, João Pedro Portilho Encide, Henrique Barbosa, Daniel da Silva De Sousa, Aldineia Pereira da Silva, Marina Sena Mendes, Maria Cristina Nonato, Albérico Borges Ferreira Da Silva, João Henrique Ghilardi Lago, Kathia Maria Honorio

**Affiliations:** 1 Center of Natural and Human Sciences (CCNH), Federal University of ABC (UFABC), Santo André, SP Brazil; 2 Faculty of Pharmaceutical Sciences (FCF), University of São Paulo (USP), São Paulo, SP Brazil; 3 São Carlos Institute of Chemistry (IQSC), University of São Paulo (USP), São Carlos, SP Brazil; 4 School of Arts, Sciences and Humanities (EACH), University of São Paulo (USP), São Paulo, SP Brazil; 5 School of Pharmaceutical Sciences at Ribeirao Preto (FCFRP), University of São Paulo (USP), Ribeirão Preto, SP Brazil

**Keywords:** Schistosomiasis, Dihydroorotate dehydrogenase, Natural products, Molecular docking, Molecular dynamics, Biological assays

## Abstract

Schistosomiasis is a parasitic disease affecting millions worldwide, highlighting the urgent need for effective treatments. This study investigated a new potential site on *Schistosoma mansoni* dihydroorotate dehydrogenase (*Sm*DHODH) and identified natural compounds as inhibitors from in silico and experimental techniques. Molecular docking and dynamics simulations revealed significant interactions between *Sm*DHODH and *ent*-kaur-16-en-19-oic acid (3) and 15β-senecioil-oxi-ent-kaur-16-en-19-oic acid (4). The complex formed by compound 3 and the biological target showed the lowest free binding energy, indicating greater stability. Experimental assays indicated that compound 3 significantly reduced *Sm*DHODH activity (40 ± 2% at 250 µM), while compound 4 had minimal effect (90 ± 1%). Structural analyses revealed that hydrophobic interactions at site 1 and conformational changes induced by compound 3 are crucial for effective enzyme inhibition. Therefore, from the computational and experimental approaches employed in this study, we were able to understand a possible mechanism related to the *Sm*DHODH inhibition and the discovery of a potential bioactive compound against this important target related to schistosomiasis.

**Supplementary Information:**

The online version contains supplementary material available at 10.1007/s40203-026-00628-1.

## Introduction

Schistosomiasis, one of the most prevalent parasitic diseases globally, affects approximately 250 million individuals worldwide (World Health Organization 2025). Current treatment relies on Praziquantel (PZQ) (Nogueira et al. 2022), which, despite being effective, has limitations including low efficacy against the immature stages of the parasite (schistosomula) and the lack of activity in preventing reinfection, which necessitates repeated treatments and raises concerns about the potential selection of resistant strains (Vale et al. 2017). Since natural products represent a significant source of scaffolds for the development of antiparasitic compounds (Azevedo et al. 2023), the search for alternative, plant-derived therapeutics remains a highly promising avenue.

An interesting approach involves targeting dihydroorotate dehydrogenase (DHODH), a flavoenzyme vital for the *de novo* pyrimidine nucleotide biosynthetic pathway. *Schistosoma mansoni* (*Sm*) relies exclusively on the salvage pathway to meet its purine needs, making *Sm*DHODH a critical therapeutic target for parasite survival (Aoki and Oya 1979; Serrao et al. 2017). Some studies have revealed key structural differences between the parasite’s DHODH and the human class 2 DHODH (Marcinkeviciene et al. 2000; Cuthbertson et al. 2020). The studies of Nonato et al. (2019) and De Mori et al. (2021) highlighted that *Sm*DHODH possesses a unique catalytic loop that directly controls the dynamics of the inhibitor binding site. This is a structural feature of significant interest for chemotherapeutic exploration (Otarigho 2019; Nonato et al. 2019), paving the way for the development of selective inhibitors.

Recent in silico efforts underscore the continued interest in this target for schistosomiasis therapy. In response to this need and the opportunity for selective inhibition, the present study conducted computational simulations to analyze the interaction between four anti-schistosomal natural products (Brito et al. 2022; Sessa et al. 2020) and the *Sm*DHODH enzyme. In addition, we also carried out biological assays and prediction of pharmacokinetic properties to identify potential bioactive inhibitors against this biological target.

## Materials and methods

### Structures of natural products

The four natural compounds (1–4, Table 1), selected for this study, were prioritized based on their previously reported anti-schistosomal efficacy in both in vitro assays and in vivo murine models (Brito et al. 2022; Sessa et al. 2020). This selection strategy was designed to bridge phenotypic efficacy with target-based characterization through *Sm*DHODH inhibition.

The three-dimensional structures of these compounds were constructed using Marvin software (ChemAxon 2024). Subsequently, the structures were prepared within the Sybyl 8.1 package (Tripos Inc. 2007), which involved the addition of hydrogen atoms and the assignment of atomic charges calculated via the semi-empirical PM3 method in the MOPAC module (Stewart 1989). Energy minimization was performed using the Tripos force field with a convergence criterion of 0.05 kcal/mol·Å to ensure stable conformational starting points for the docking simulations. The chemical structures of the studied compounds and the positive control, Praziquantel (Nogueira et al. 2022), are presented in Table 1. Detailed biological activity data, including lethal and effective concentrations (LC_50_ and EC_50_), are available in the Supplementary Information (Table S1).

**Table 1:** Structure of compounds 1–4 and the positive control (Praziquantel - PZQ) used in this study

Compound	Chemical structure	Name
1		Threo-austrobailignan-6
2		Verrucosin
3		*Ent*-kaur-16-en-19-oic acid
4		15β-senecioil-oxi-*ent*-kaur-16-en-19-oic acid
PZQ		Praziquantel

### Preparation of protein structure and study of binding site

The crystal structure of the *Sm*DHODH enzyme was obtained from the Protein Data Bank (PDB) with ID 6UY4 (de Mori et al. 2021). This structure has a resolution of 2.80 Å and presents the co-crystallized ligand QLA (2-[(4-fluorophenyl)amin]-3-hydroxynaphthalene-1,4-dione). The enzyme structure was prepared for docking simulations using the Sybyl package, which involved removing water molecules, adjusting poor contacts, and adding hydrogen atoms and charges. To validate the selection of *Sm*DHODH, using the SwissPredictTarget server (Daina et al. 2019), we predicted potential biological targets with which the four selected compounds could interact based on the results displayed in Supporting Information (Fig. S2).

The iMODS (López-Blanco et al. 2014), FTmap (Kozakov et al. 2015), and CavityPlus (Wang et al. 2023) servers were used to search for potential allosteric sites, as well as to map these cavities for molecular docking. The iMODS server was employed to analyze the apo form’s structural movements and to assess flexibility and correlated regions. Subsequently, using FTmap we identified druggable hot spots by mapping the surface with organic probes, while CavityPlus was used to obtain the geometric and physicochemical properties of these pockets. This integrative approach ensures a robust selection of viable binding sites.

### Molecular docking and studies of ligand-receptor interactions

We performed docking simulations with GOLD software (Jones et al. 1997). Initially, redocking was carried out at the site of inhibitor (site 1) to validate docking parameters, using the Chemscore function, a 5 Å radius, and Ser53 as a flexible residue for correct ligand orientation. For the simulations considering the inhibitor´s binding site (site 1) and the potential new site (site 2), the FMN cofactor was maintained to preserve the integrity of the enzyme. In the new potential site (site 2), the docking simulations were centered on Thr273 or Asn272 within a 7 Å radius, using the same scoring function (Chemscore) without flexibility adjustments.

### Prediction of ADMET properties for the selected compounds

ADMET (absorption, distribution, metabolism, excretion, and toxicity) properties of the studied molecules were predicted using the SwissADMETox (Bueno 2020) and MolInspiration (Molinspiration Cheminformatics 2025) servers.

### Lipinski rules

Values for solubility, hydrogen bond donors and acceptors, molecular weight, drug-likeness, and drug score of each molecule under study were also calculated. Subsequently, the same molecules were analyzed on the Molinspiration server, which allowed for the analysis of the topological polar surface area (TPSA) and the estimation of oral absorption percentages for all compounds studied. This methodology is based on Lipinski’s Rule of Five, which can help to predict the compound’s oral bioavailability, ensuring that the molecule possesses properties that would make it likely to be an orally active drug in humans.

### Molecular dynamics (MD) simulations

After the docking analyses, the top-ranked ligand-protein complexes (considering sites 1 and 2) were selected for molecular dynamics (MD) simulations using Amber18. Topology files were generated with the tLEaP module (Ambertools18) (Case et al. 2005), employing the ff99SB force field. The molecular systems were solvated in an octahedral box with TIP3P water, neutralized with sodium and chloride ions, and equilibrated in ten incremental steps (0–300 K) totaling 13 ns. Then, we carried out production runs by using time step of 1 fs for 120 ns long MD simulations (corresponding to 120,000 frames).

The RESP (Restrained Electrostatic Potential) charges of ligands were calculated at HF/6-31G* level. Finally, the RMSD (Root Mean Square Deviation) and RMSF (Root Mean Square Fluctuation) analyses were performed using the CPPTRAJ module. The most stable trajectory was used to calculate the ∆G value related to the ligand-receptor complexes. All MD simulations were performed at the HPC (High-Performance Computing) at the University of São Paulo using Amber18 software.

### Calculations of free binding energy

From the MD simulations, parameters related to the free binding energy were calculated. For this purpose, we employed the SIETRAJ program (Naim et al. 2007) that uses the Poisson–Boltzmann equation and the boundary element method (BEM) (Bosy et al. 2024). The complexes formed by compounds and the biological receptor, taking account both binding sites, were used to estimate the free binding energy. These analyses aimed to assess the stability of the compounds at the DHODH sites and enhance the robustness of this study.

### Experimental assays

To evaluate the inhibition of the two most promising natural products (compounds 3 and 4) against *Sm*DHODH, selected from the in silico analyses, a continuous colorimetric assay was performed. In this assay, the reduction of DCIP (2,6-dichloroindophenol) was monitored at 610 nm for 60 s at 25 °C, using the SpectraMax 384 Plus microplate reader (Devices). For the enzymatic reaction, 190 µL of the reaction mixture was used, consisting of 50 mM Tris buffer (pH 8.15), 150 mM KCl, 0.1% Triton X 100, 1 mM L-dihydroorotate, 18 µM CoQ0, and 60 µM DCIP. The reaction was initiated by adding 10 µL of *Sm*DHODH, achieving a final concentration of 40 nM. Inhibition assays were started with a single-point test using the tested compounds at 250 µM in the reaction mixture, with a 2 hour incubation of the protein with the compound. Each compound was tested in triplicate. DMSO was added to the reaction mixture as a reference, without inhibitors. IC_50_ assays were performed under the same experimental conditions as the single-point tests but with inhibitor concentrations ranging from 250 to 0.03 µM. The IC_50_ value was determined from the inhibition percentage versus log inhibitor concentration graph. The dose-response curve was fitted according to Eq. 1 using GraphPad Prism 5 software (GraphPad Software 2007).

1 $${\mathrm{y}} = \frac{{100}}{{1 + 10^{{x - \log {\mathrm{IC}}50}} }} $$

where y represents the percentage of residual activity, ranging from 100% (no inhibition) to 0% (total inhibition), x corresponds to the decimal logarithm of the inhibitor concentration, and IC_50_ corresponds to the concentration that reduces enzyme activity by 50%.

## Results and discussion

### Structural and molecular docking studies

**Fig. 1:**
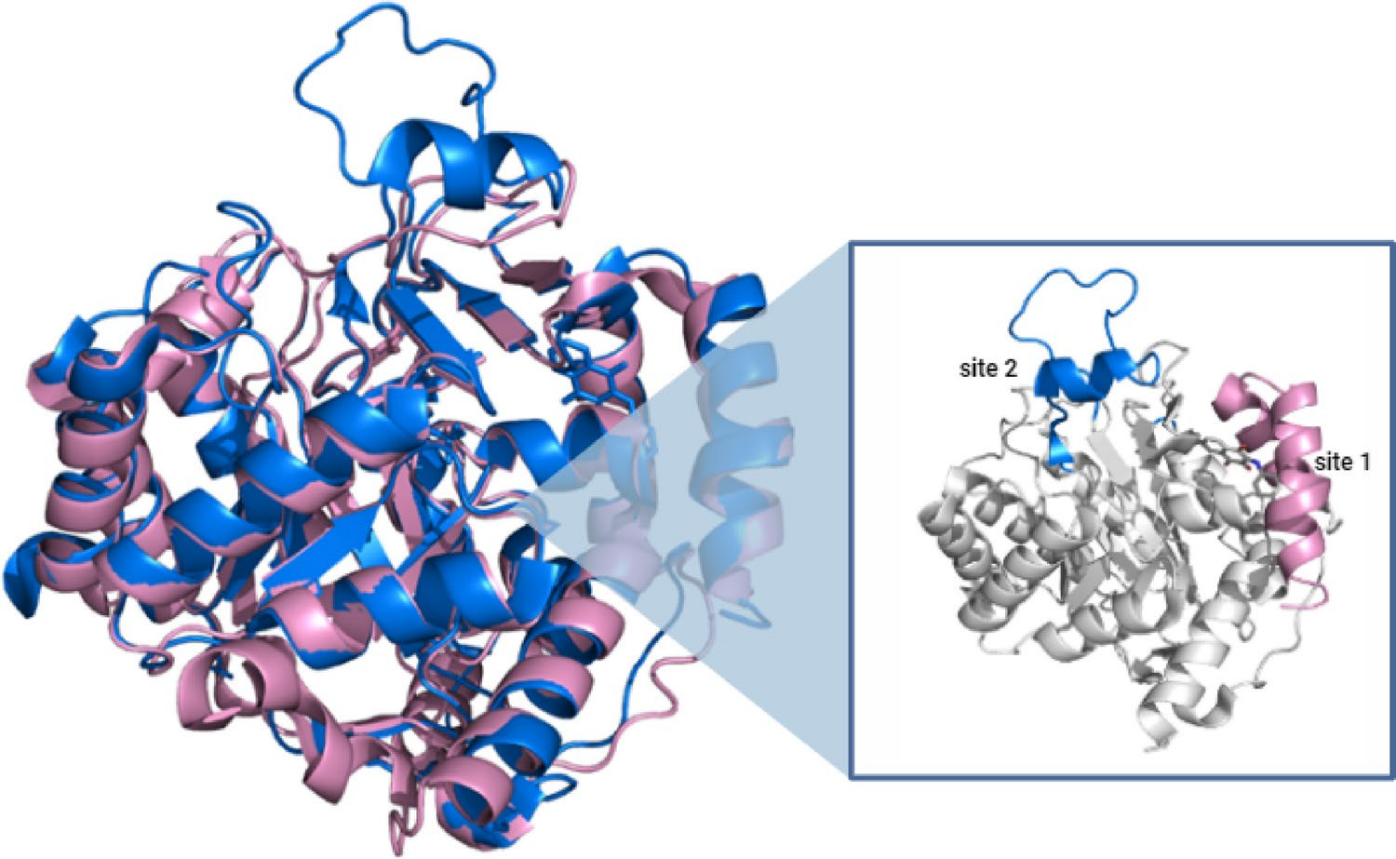
Overlay of the human DHODH enzyme and the *S. mansoni* enzyme to identify differences between the human (pink) and parasite (blue) structures. The protruding blue loop at the top of the structure corresponds to the potential site of *Sm*DHODH, which may coordinate the entry of compounds into the binding pocket. The presence of ten residues (Gly285 to Lys294) in the loop of binding site 2 (highly conserved across all species of the Schistosoma genus) allows for significant rearrangement in this region compared to other classes of DHODH structures

Figure 1 shows the overlay of the human DHODH enzyme (*Hs*DHODH) (PDB code: 2FPV) (Baumgartner et al. 2006) in pink and the *S. mansoni* DHODH enzyme (*Sm*DHODH) in blue. Despite the overall similarity to the human enzyme, substitutions and differences in the conformational states of *Hs*DHODH and *Sm*DHODH structures may allow for the identification of selective inhibitors for the parasite enzyme, such as the loop region in the upper area of *Sm*DHODH that is absent in the human enzyme and may indicate access to a potential site.

**Fig. 2:**
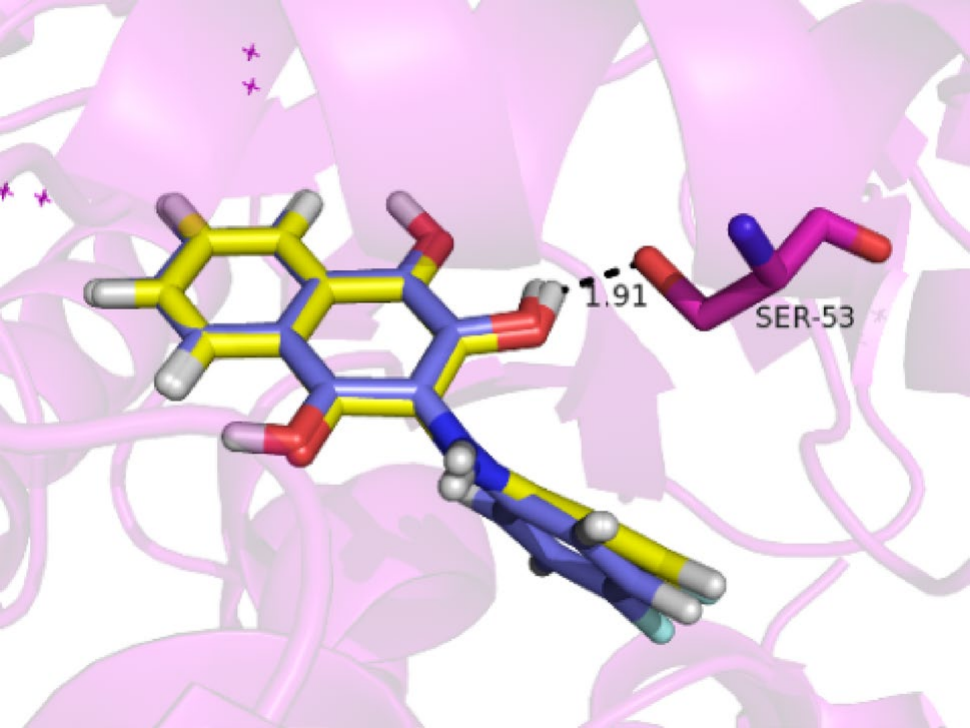
Superposition of QLA from the docking simulations and the experimental conformation of the compound within the active site of the biological receptor. The experimental conformation (from the PDB structure) of the crystallographic ligand is shown in yellow, while the pose obtained from docking is represented in blue. Ser53 was made flexible to coordinate the hydroxyl group on the central ring, as predicted by the experimental assays

Figure 2 presents the redocking results for the crystallographic ligand (QLA) at the binding site 1 of the target. From the results, we can see that the selected docking parameters (Chemscore function, radius of 5 Å, Ser53 as flexible residue) successfully reproduced the experimental conformation (RMSD value of 0.417 Å).

### Comparison of binding sites

#### Analysis of site 1 (inhibitor´s binding site)

**Fig. 3:**
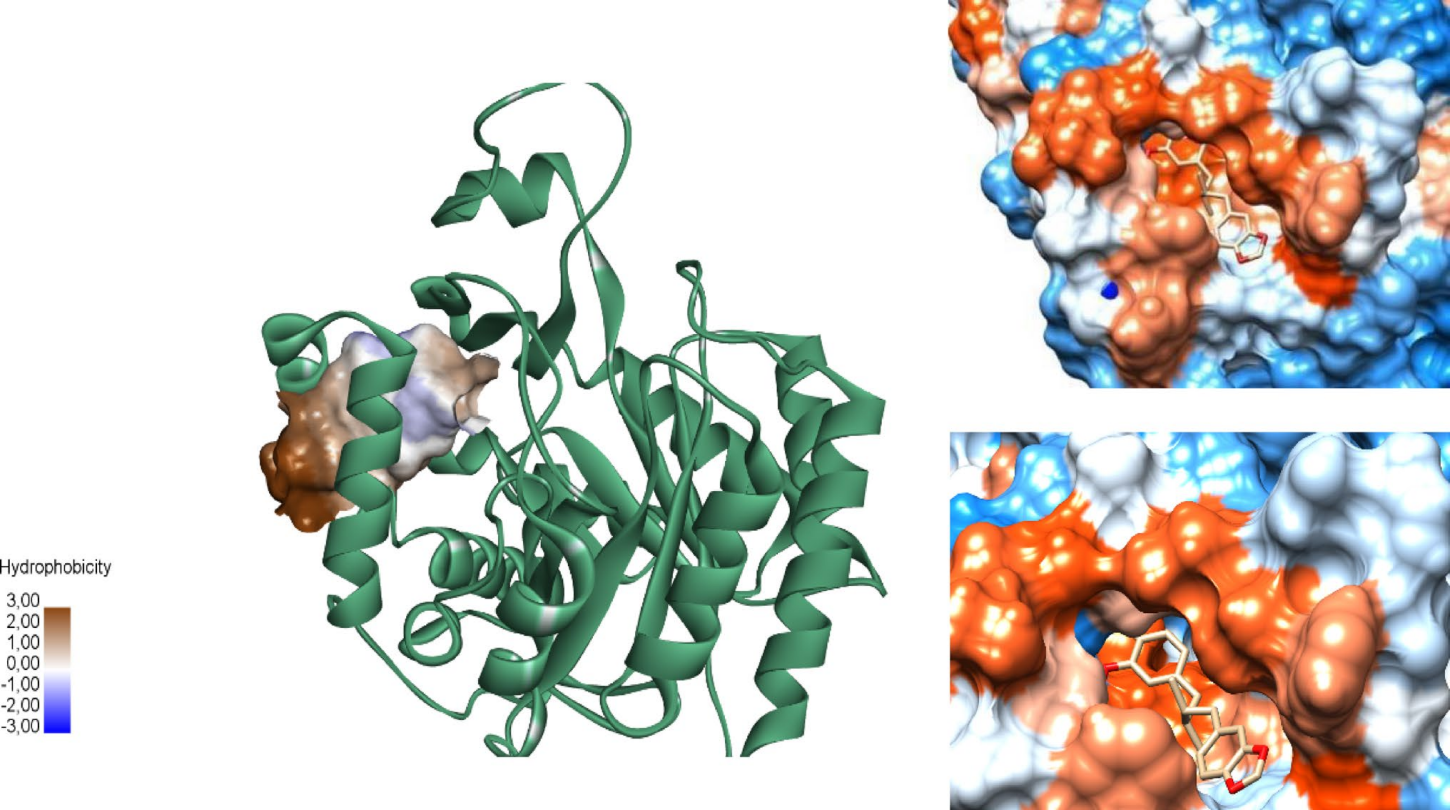
Hydrophobic profile of the site 1 with the crystallographic ligand. The site’s entrance presents residues with hydrophobic characteristics (orange); polar residues (blue) form the interior of the pocket. The crystallographic ligand is located in the orange region at the entrance of the binding site

Using the FTSite server, a radius of 5 Å around the ligand was selected within the two binding sites under analysis. The most common probes found in the interior of site 1 were DME (dimethyl ether) and THS (isopropanol), which characterize polar interactions and hydrogen bond acceptors. Figure 3 shows an intense orange color at the inhibitor site’s entrance, representing hydrophobic residues where the crystallographic ligand is retained. Additionally, Fig. 3 displays a blue region within the interior of site 1, where the probes characterizing polar interactions are located, which is necessary to understand the interaction with the studied compounds.

#### Analysis of site 2 (potential binding site)

**Fig. 4:**
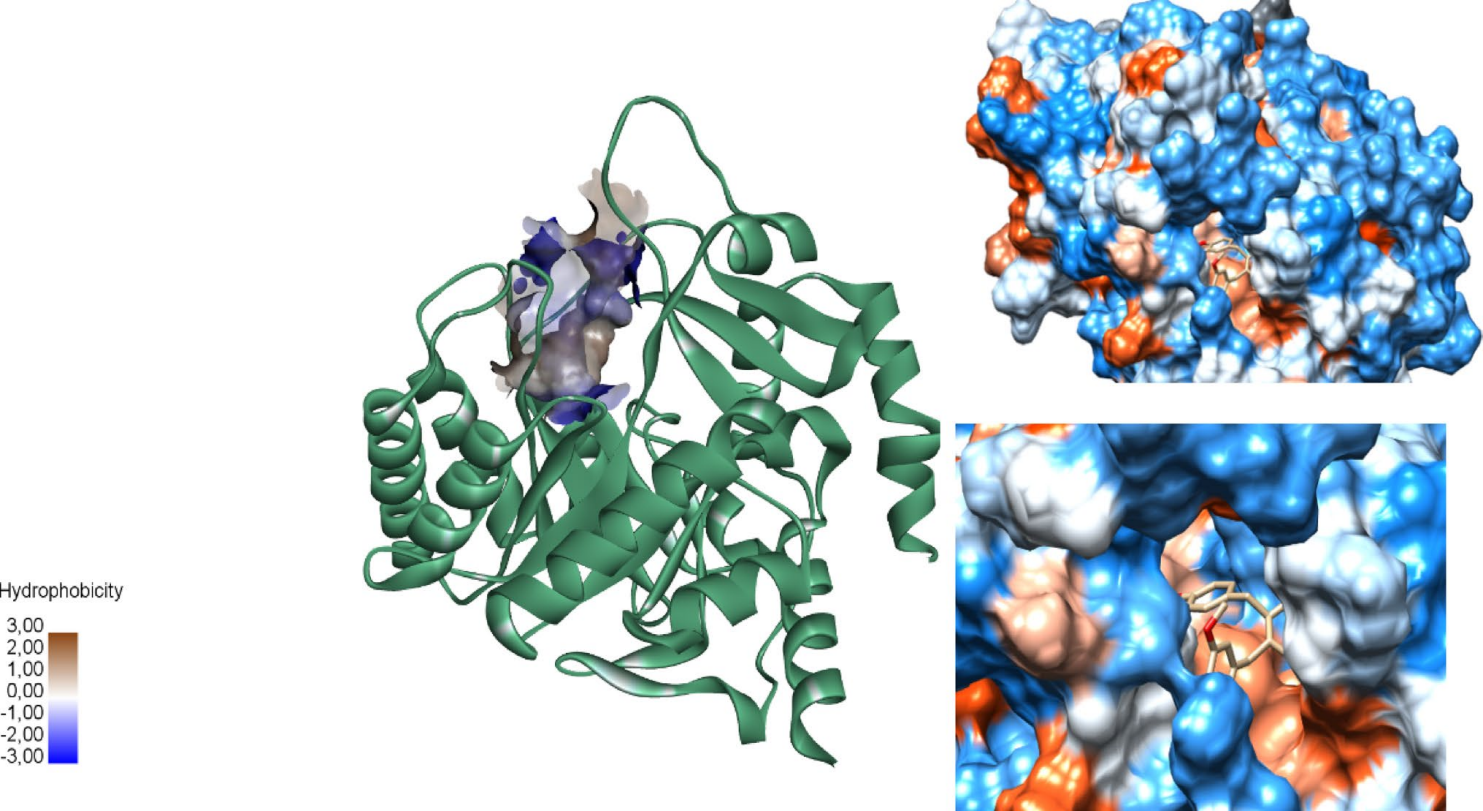
Hydrophobic profile of the studied potential site (2). The loop at the entrance of the binding site shows residues with hydrophilic features (Pro290, Ile289, and Val297) that could coordinate the entry of the drug. Inside the pocket, there are several residues with apolar profile

In the entrance of site 2, loops exhibit significant flexibility in the opening and closing of the pocket. In the outermost region, the loops were predominantly hydrophobic. However, as they approach the pocket region, the loops displayed a hydrophilic character (blue region in Fig. 4). Moving further into the site, hydrophobic residues reappeared in greater abundance. The most frequent probes observed in the characterization of site 2 were BUT (tert-butanol), CHX (cyclohexane), DFO (N, N-dimethylformamide), and PHN (phenol). The BUT probe is hydrophobic and aromatic in the pocket’s innermost region (Fig. S3).

In contrast, the polar probes were located at the entrance of the flexible loop and exhibited hydrogen bond donor and acceptor characteristics. Figure 4 shows site 2 with its hydrophilicity surface. These findings align with other studies in the literature (Nonato et al. 2019; de Mori et al. 2021), which suggest that the high flexibility of the protruding loop is a crucial regulator of the catalytic activity of the biological receptor.

### Structural analyses of binding sites

The results obtained from the CavityPlus server identified multiple clusters representing potential cavities in the *Sm*DHODH receptor. Cluster 1 stood out due to its significant volume (2432.50 Å^3^) and surface area (1905.00 Å^2^). This cavity is particularly interesting as it established a functional connection between sites 1 and 2, suggesting that the cofactor between these sites might mediate simultaneous interactions with both. This structural configuration indicates a possible active role of the cofactor in stabilizing the conformations necessary for the enzymatic activity, potentially influencing both substrate binding and catalysis. The most relevant residues in these interconnected pockets included Asp45, Asn123, Pro126, Ser271, Arg130, and Tyr354 (the complete residue list is available in Supporting Information Table S4).

**Fig. 5:**
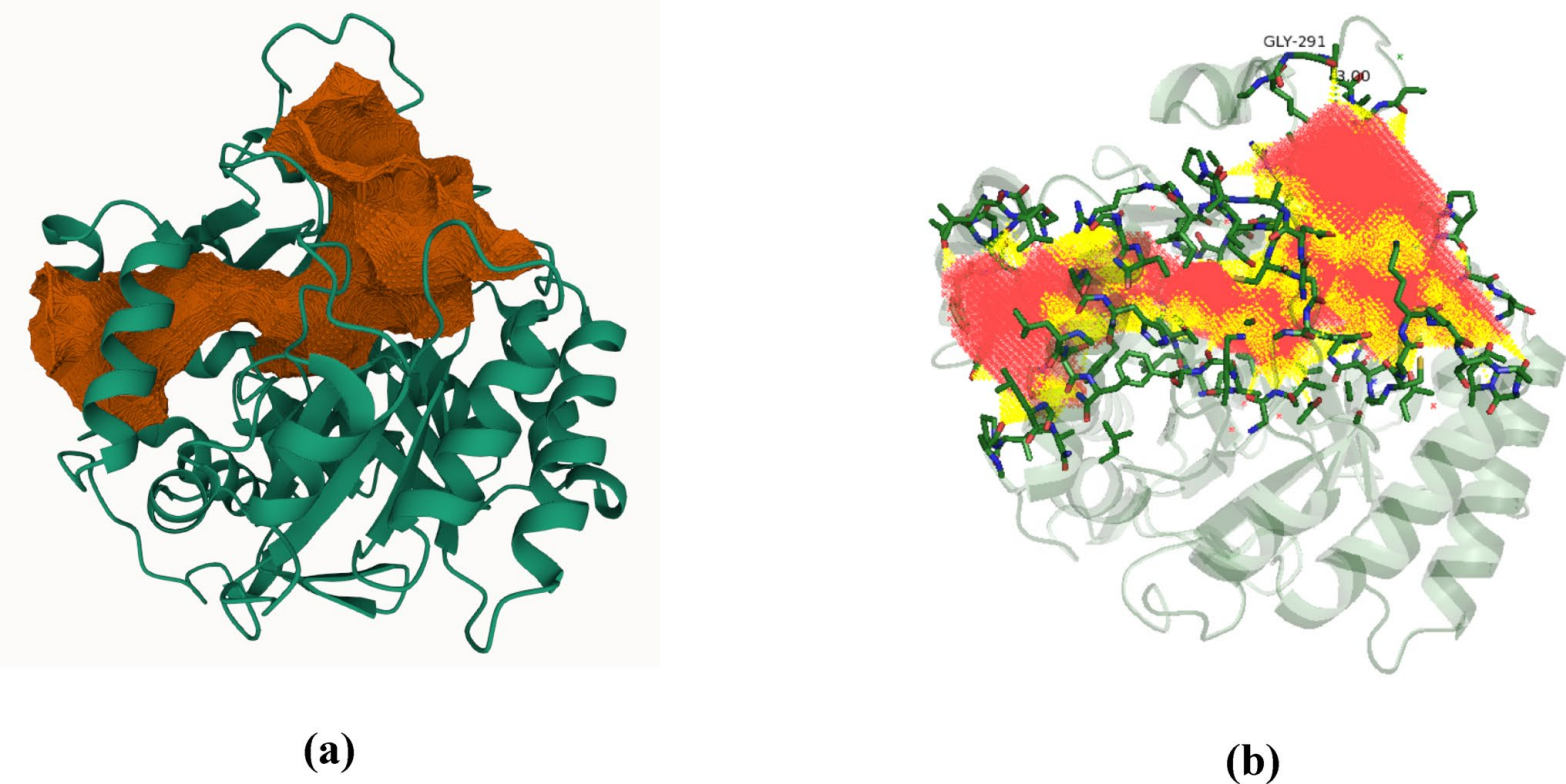
**a** Three-dimensional representation of the *Sm*DHODH structure with emphasis on cluster 1. The regions in green correspond to the protein structure, while the orange areas represent the cavities identified from the analysis. Cluster 1 shows a connection between the active sites, suggesting potential interactions between these locations. **b** The yellow regions represent polar contacts of the binding sites, and the red regions indicate hydrophobic contacts.

Figure 5a shows the volume of cluster 1, while Fig. 5b highlights the regions of the cluster with polar (yellow) and apolar (red) contacts. We observed that both sites’ (1 and 2) entrances are predominantly apolar. However, as one moves further into site 2, there is a significant concentration of polar contacts, suggesting a capability to stabilize compounds with amphiphilic characteristics, enabling interactions with hydrophobic and hydrophilic regions

the docking results of the selected compounds at site 2

### Analyses of movements and flexibility of the target structure

**Fig. 6:**
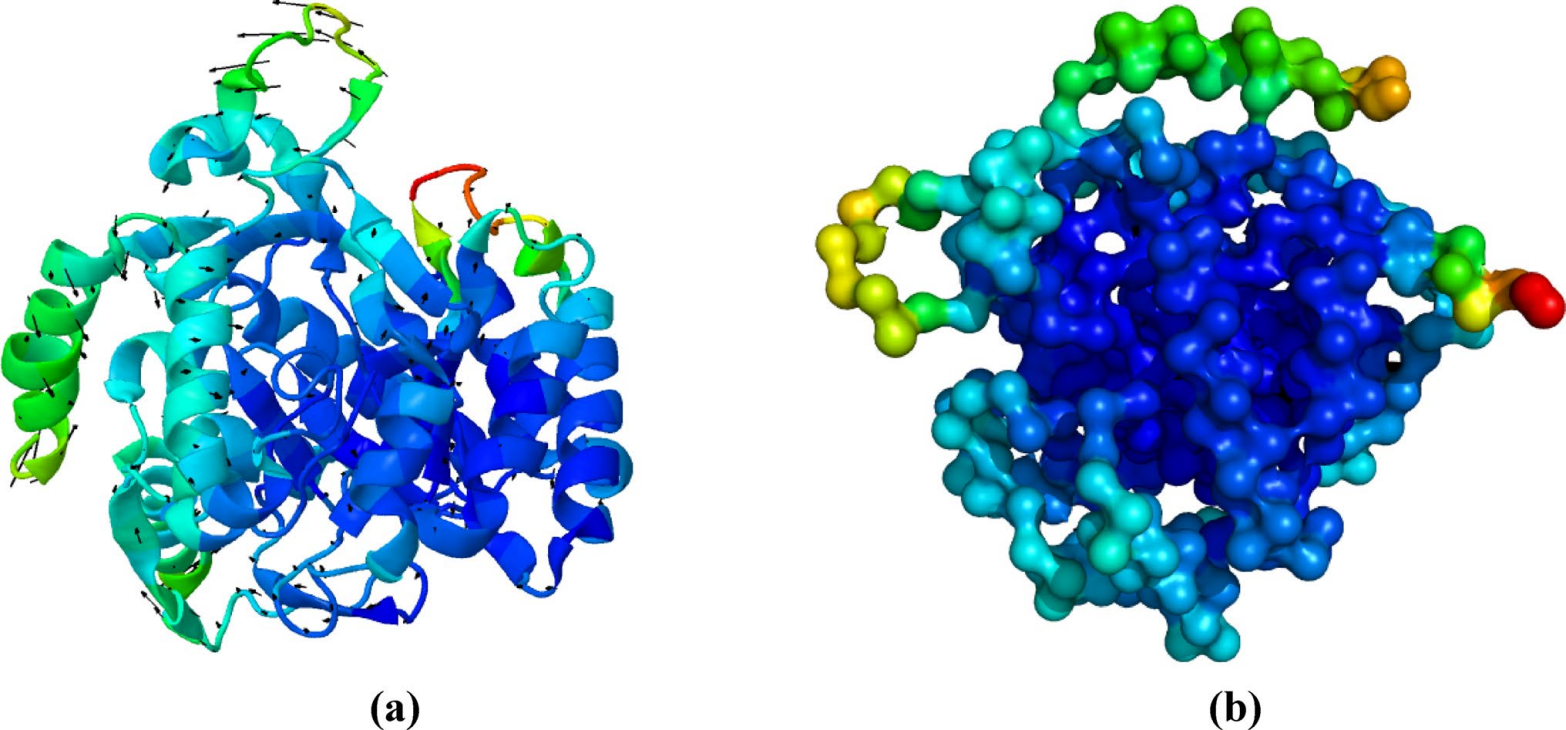
**a** Structure of the enzyme with the field of arrows, using mode 5, which show twisting or rotational movements between domains, domain bending, and global opening and closing movements; **b** representation of *Sm*DHODH in ball and stick model. Blue: regions with low B-factor values, meaning that the atoms in this area are more ordered or have lower mobility. Green and yellow: intermediate B-factor values. Red: regions with high B-factor values, suggesting more flexible or disordered atoms

To understand the enzyme’s global movements and its flexibility properties, we employed the iMODS server (López-Blanco et al. 2014) to analyze the structural dynamics of the apo form of *Sm*DHODH. The distribution of the B-factor, which reflects atomic mobility, showed intermediate values (yellow and green colors, Fig. 6a and b) in both sites (1 and 2), indicating a correlation in the flexibility and movements of these regions. The normal mode analysis provided essential insights into this structural flexibility: the vectors indicated that the loop at site 1 exhibits a vertical movement (suggesting an opening/closing mechanism) and the loop at site 2 showed a twisting movement, which influences the overall enzyme conformation and may regulate access to the binding pocket.

**Fig. 7:**
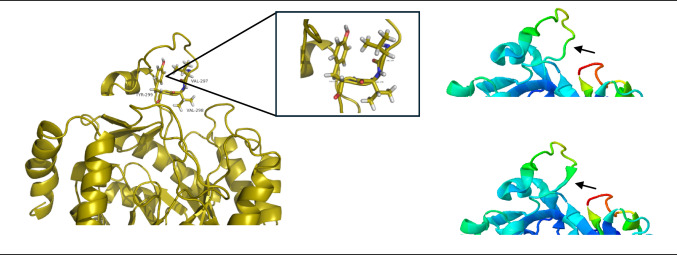
Residues at the loop related to the global movements of the enzyme and zoom of the region related to the loop movement involving Val297, Val298, and Tyr299

Additionally, the analysis of normal modes revealed that the movement of Tyr299 is crucial for the enzyme regulation. The position of the Tyr299 side chain alternated between staying in the plane and causing steric hindrance, potentially preventing the loop from closing further over site 2 (Fig. 7). This intrinsic flexibility, particularly the movement of Tyr299 that sterically modulates site 2, is a key structural characteristic supporting the subsequent hypothesis of the potential site´s modulation by compound 3.

The eigenvalues showed in Fig. S5a (Supporting Information) indicate the rigidity associated with each mode of movement; higher eigenvalues imply modes requiring greater energy for deformation, thus being more rigid. Additionally, Fig. S5b displays cumulative variance, indicating the relative impact of each mode on the enzyme’s overall mobility. Modes with lower eigenvalues exhibit higher variance and significantly contribute to structural flexibility.

### Docking simulations at the inhibitor binding site (1)

**Fig. 8:**
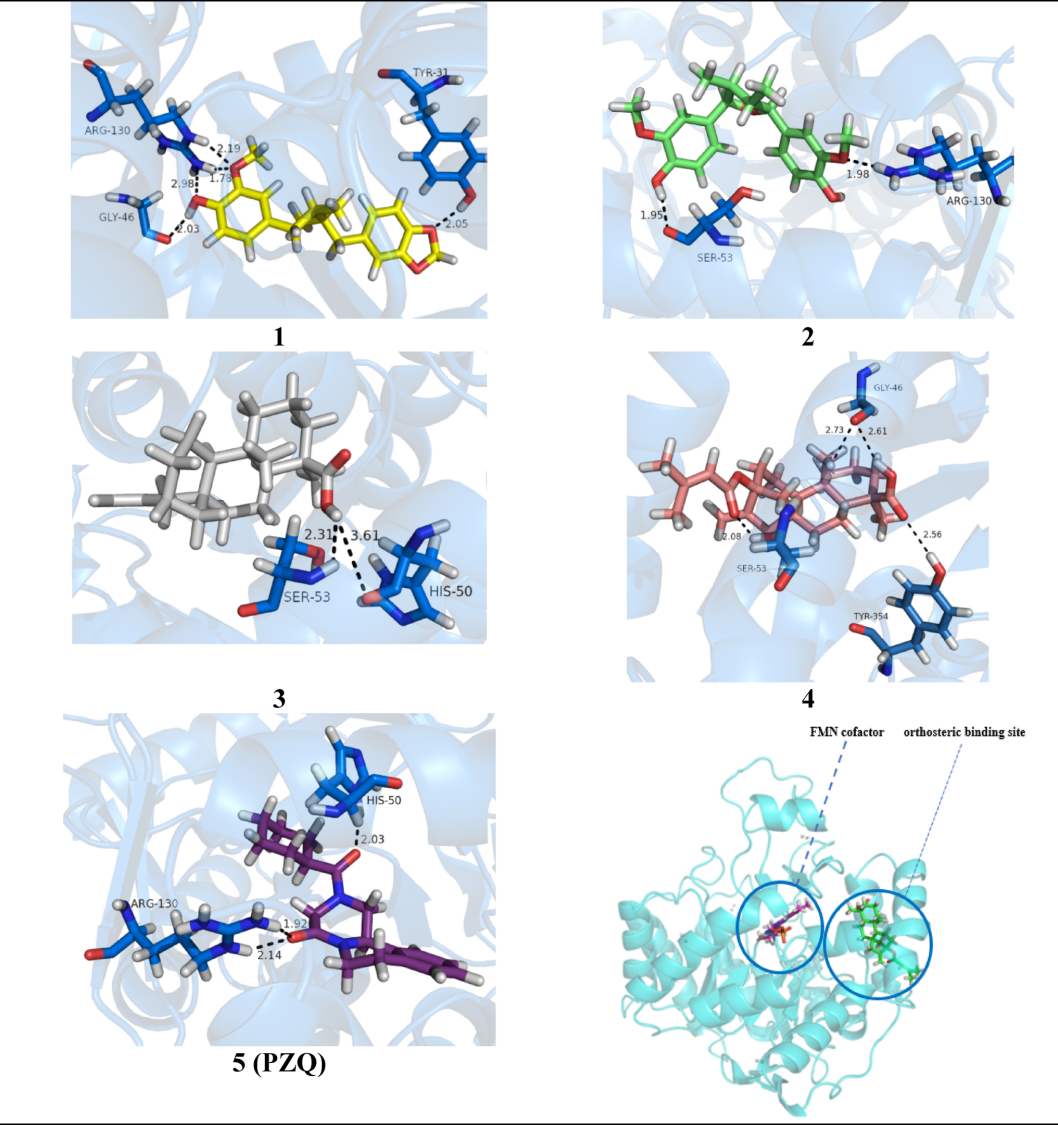
Results of the compounds docked at the biological receptor (*Sm*DHODH) with the best poses obtained from the docking simulations at site 1, emphasizing hydrogen bonds with the residues in the selected site

Figure 8 presents the docking results related to the selected compounds at the site 1 of the biological target. Table 2 provides key information obtained during the ligand-protein simulations, such as the estimated energy values and observed interactions.

**Table 2:** Key results obtained from the docking simulations characterizing the interactions of the compounds at the enzyme’s binding site 1

Compound	Binding interactions		No of interactions (H-bond)	Interaction types (residues)		
	ΔG (kcal/mol)^a^	Fitness^b^		H-bonds	vDW^c^	π-Alkyl
1	− 37.89	34.32	6	Tyr31 Arg130 Gly46	Phe35 Ala39 Ser53 His50 Gly361	Ala49 Val358 Pro362
2	− 35.31	24.99	2	Ser53 Arg130	Gly361 Ala57 Tyr31 Phe357 Phe92 His50	Ala49 Val358 Pro362
3	− 33.69	24.25	2	His50 Ser53	His50 Pro362 Leu56 Tyr31 Phe35 Gly361	Leu36 Phe357
4	− 38.70	24.78	4	Tyr354 Gly46 Ser53	Ile128 Gly46 Glu47 Val43 Leu56 Arg40	Val358 Ala49
PZQ	− 36.78	33.85	3	Arg130 His50	Phe35 Tyr31 Ser53 Phe92 Tyr354 Val137 Gly46	Leu36 Ala39 Ala49 Leu128

a = Gibbs free energy (ΔG) is calculated as a sum of these terms, and the final ChemScore value used to indicate binding affinity. Some of the terms that can be considered in the ChemScore function include: Binding energy terms = van der Waals energy, and electrostatic energy; Shape complementarity terms = steric overlap and volume terms; Charge complementarity terms evaluate the charge complementarity between the ligand and the protein’s active site; Desolvation scoring terms assess the energy cost associated with the desolvation of the ligand and the protein’s active site during the formation of the ligand-protein complex; b = fitness of a scoring function measures how well it distinguishes between ligand poses likely to represent true interactions and poses that are artifacts or irrelevant

Compound 4 exhibited the lowest LC_50_ value (11.6 µM, Table S1), indicating a lower concentration required to cause death in 50% of the tested cells. This compound also displayed the best performance in the docking simulations, with a more negative ΔG of − 38.70 kJ/mol, indicating a strong interaction with the inhibitor´s binding site of the target protein. Additionally, compound 4 presented four hydrogen bonds, six van der Waals interactions, and two π-alkyl interactions with some important residues, suggesting good structural complementarity with the protein’s active site.

Compound 3 (26.1 µM, Table S1) exhibited only two hydrogen bonds and three π-alkyl interactions with important residues at the binding site. However, the hydrophobic interactions with the apolar portion of the compound proved to be decisive for the stability of the complex. These interactions mainly concentrated around the central hydrophobic ring and the senecioyl moiety, significantly contributing to the molecule’s fitting into the cavity.

### Docking simulations at the potential binding site (2)

**Fig. 9:**
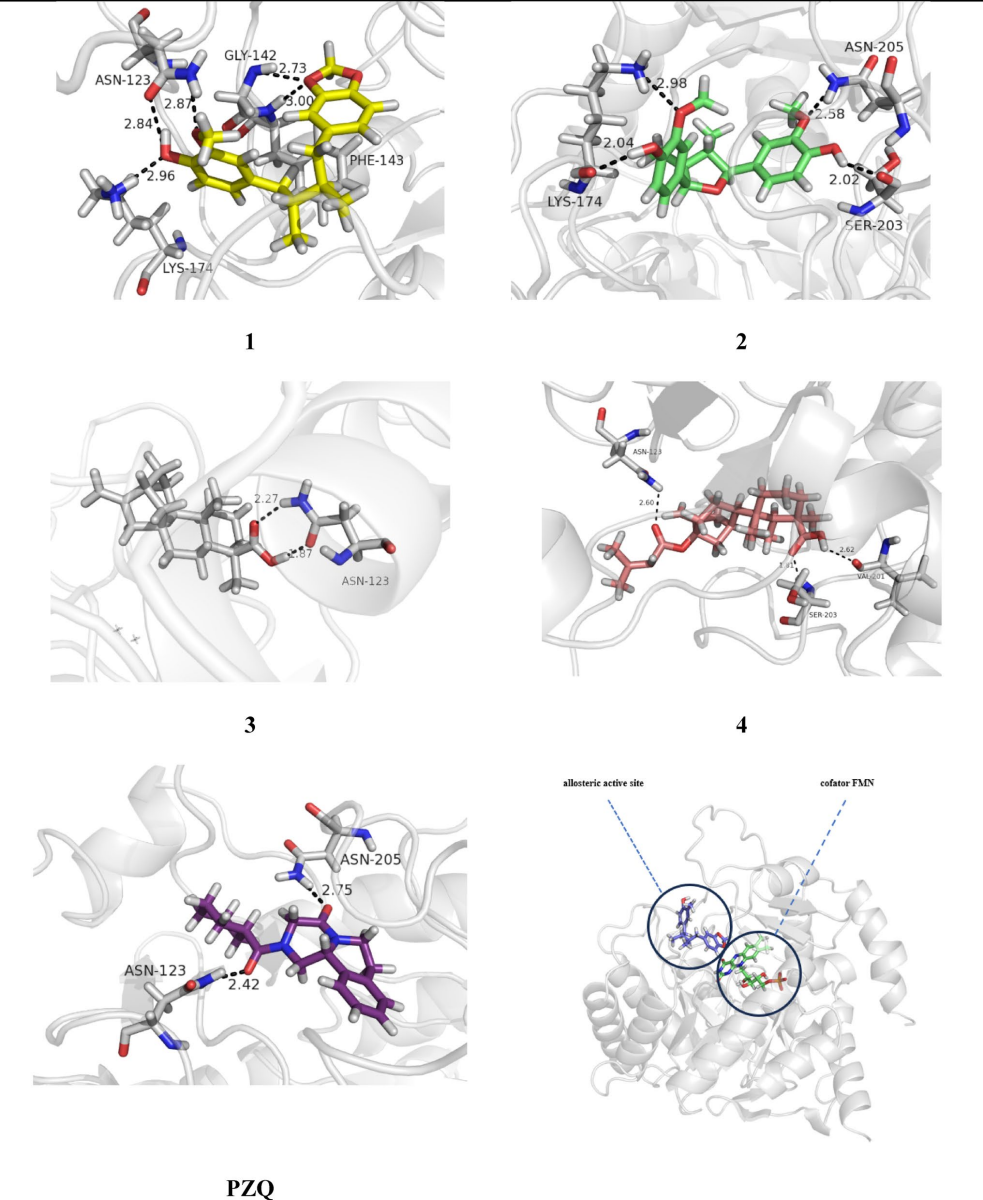
Results of the compounds 1–4 and PZQ docked to the biological receptor (*Sm*DHODH) with the best poses obtained at the potential binding site. Hydrogen bonds with residues at site 2 are highlighted

Figure 9 presents the docking results of the selected compounds at site 2; Table 3 shows the key ligand-protein interactions. The potential binding site (2) presented an external loop composed of hydrophobic residues, followed by a polar region at the entrance and another internal hydrophobic region. Compounds 2 and 4 exhibited the best interaction energies (see Table 3), notably forming hydrogen bonds with Asn205 and Ser203. The methoxyphenol group of compound 2 formed four hydrogen bonds with Lys174, Asn205, and Ser203, suggesting a potential blockade of access to the internal hydrophobic region. Compound 4 established hydrogen bonds with Ser203, Val201, and Asn123 from its ester and carboxylic acid groups.

All compounds displayed π-alkyl interactions regarding hydrophobic interactions, with PZQ presenting the highest number. Compound 2 exhibited the second-best biological activity and the highest number of π-alkyl interactions, facilitating its stabilization within the site. Compound 3 made a hydrogen bond with Asn123 and exhibited fewer hydrophobic interactions. Analyzing interactions at site 2 may provide insights for potential new modulation of this biological target. Previous studies (de Mori et al. 2021; Nonato et al. 2019) suggest that the occupancy of site 1 modulates the movement of the catalytic loop near site 2, impairing the redox reactions occurring at the orthosteric site.

### Prediction of ADMET properties of the compounds

Table 4 presents the predictions from the SwissADMETox (Bueno 2020) server regarding the pharmacokinetic/toxicity viability of the five compounds under study. The prediction results help to assess the pharmacokinetic profile and toxicity of the compounds, as it directly influence the bioavailability of each compound in the body.

Table 4 shows that compounds 1–4 and PZQ exhibited high-predicted intestinal absorption (92.5–96.2%), indicating efficient gastrointestinal uptake. Aqueous solubility values ranged from − 3.56 to − 4.70 (log mol/L), with all compounds having moderate to good solubility; PZQ was the least soluble. Regarding blood-brain barrier (BBB) permeability, only PZQ was predicted to moderately cross into the central nervous system (CNS), while other compounds would likely remain excluded. Analyzing the metabolism data, compounds 1, 3, 4, and PZQ were identified as CYP3A4 substrates, suggesting hepatic metabolism and potential influence on drug half-life. Only PZQ was predicted as a CYP2D6 inhibitor, which could influence the metabolism of other co-administered drugs. Regarding elimination, compounds 1 and PZQ were classified as renal OCT2 transporter substrates, suggesting predominant renal clearance.

**Table 3:** Key results obtained from the docking simulations of the selected compounds (1–4) and PZQ at site 2 of *Sm*DHODH

Compound	Binding interactions			No of interactions (H-bond)	Interaction types (residues)		
	ΔG (kcal/mol)^a^		Fitness^b^		H-bonds	vDW^c^	π-Alkyl
1	− 38.39	38.26		5	Gly142 Phe143 Asn123 Lys174	Ser202 Thr206 Gly208 Gln120 Asn139 Asn144 Lys94 Asn272	Lys174
2	− 40.95	36.50		4	Ser203 Asn205 Lys174	Gly120 Gly142 Val298 Ser202 Thr206 Asn173	Gly208
3	− 30.52	29.49		2	Asn123	Gly208 Thr206 Asn205 Ser203 Ser202 Gly142	Phe143
4	− 39.70	35.32		3	Asn123 Ser203 Val201	Thr206 Lys174 Gly208 Leu209 Ser202 Asn200 Asn272 Asn205 Gly142 Val297	Phe143
PZQ	− 37.60	32.77		2	Asn205 Asn123	Phe92 Tyr354 Val137 Gly46 Ser53 Phe357 Pro362	Leu36 Ala39 Ala49 His50

a = Gibbs free energy (ΔG) is calculated as a sum of these terms, and the final ChemScore value used to indicate binding affinity. Some of the terms that can be considered in the ChemScore function include Binding energy terms: van der Waals energy, and electrostatic energy; Shape complementarity terms: steric overlap and volume terms; Charge complementarity terms indicate the charge complementarity between the ligand and the protein’s active site; Desolvation scoring terms are related to the energy cost associated with the desolvation of the ligand and the protein’s active site during the formation of the ligand-protein complex. b = fitness of a scoring function measures how well it distinguishes between ligand poses likely to represent true interactions and poses that are artifacts or irrelevant

**Table 4:** Prediction of pharmacokinetic/toxicity (ADMET) properties of the studied compounds

Cpd	Absorption		Distribution		Metabolism		Excretion		Toxicity	
	Intestinal absorption (human) % absorbed	Water solubi-tily (log mol/L)	BBB permea-bility (log BB)	CNS permea-bility (log PS)	CYP3A4 substrate	CYP2D6 Inhibitor	Total clearance (log ml/min/kg)	Renal OCT2 substra-te	AMES	Hepa-totoxi-city
1	92.54	− 4.70	− 0.41	− 1.44	Yes	No	− 0.02	Yes	No	No
2	93.92	− 4.25	− 0.13	− 0.07	No	No	0.02	No	No	No
3	96.28	− 4.55	− 0.14	− 1.67	Yes	No	0.50	No	No	No
4	93.45	− 4.59	− 0.29	− 1.48	Yes	No	0.37	No	No	No
PZQ	93.87	− 3.56	0.17	− 0.50	Yes	Yes	1.18	Yes	No	No

### Molecular dynamics (MD) simulations

After analyzing the ADMET properties and molecular docking results, the next stage of this study involved MD simulations for a more robust evaluation of the stability of the ligand-protein complexes. Therefore, the five complexes formed between the studied molecules and the *Sm*DHODH enzyme (considering the two previously binding sites) were simulated with the Amber18 software.

**Fig. 10:**
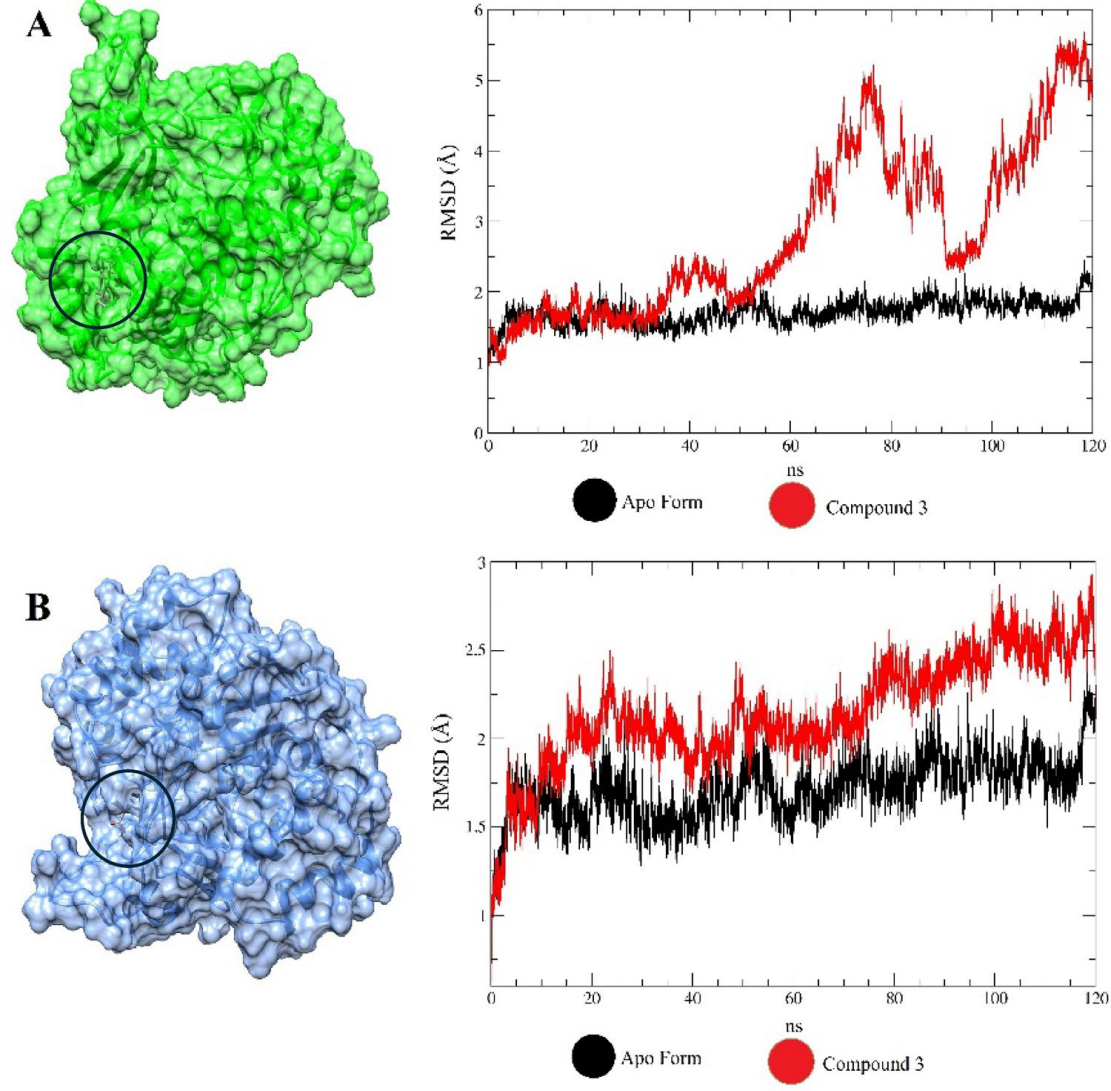
RMSD plots for the complexes formed between compound 3 and binding sites 1 (**a**) and 2 (**b**). The black lines represent the apo form of *Sm*DHODH, while the red lines correspond to the complexes formed with compound 3 at sites 1 (**a**) and 2 (**b**)

The first MD analysis involved the evaluation of the RMSD values for the ligand-protein complexes. Figure 10 shows the RMSD plot for the complex formed between compound 3 and sites 1 and 2 of *Sm*DHODH. The results for the other complexes are available in Supporting Information (Fig. S6 a, b, c and d). From Fig. 10 (RMSD plot), we can see that, at site 1, the binding of compound 3 to *Sm*DHODH induced a significant conformational change, which is intensified between 60 ns and 110 ns of simulation. This structural change contrasts with the apo form, which remains more stable. Additionally, the residue fluctuation analysis (RMSF) revealed that the region between residues 150 and 200 exhibited greater mobility in the presence of compound 3, suggesting that its binding to site 1 induced a conformational change that may propagate toward site 2. This potential conformational variation between the sites could be crucial for blocking the catalytic activity of *Sm*DHODH.

**Fig. 11:**
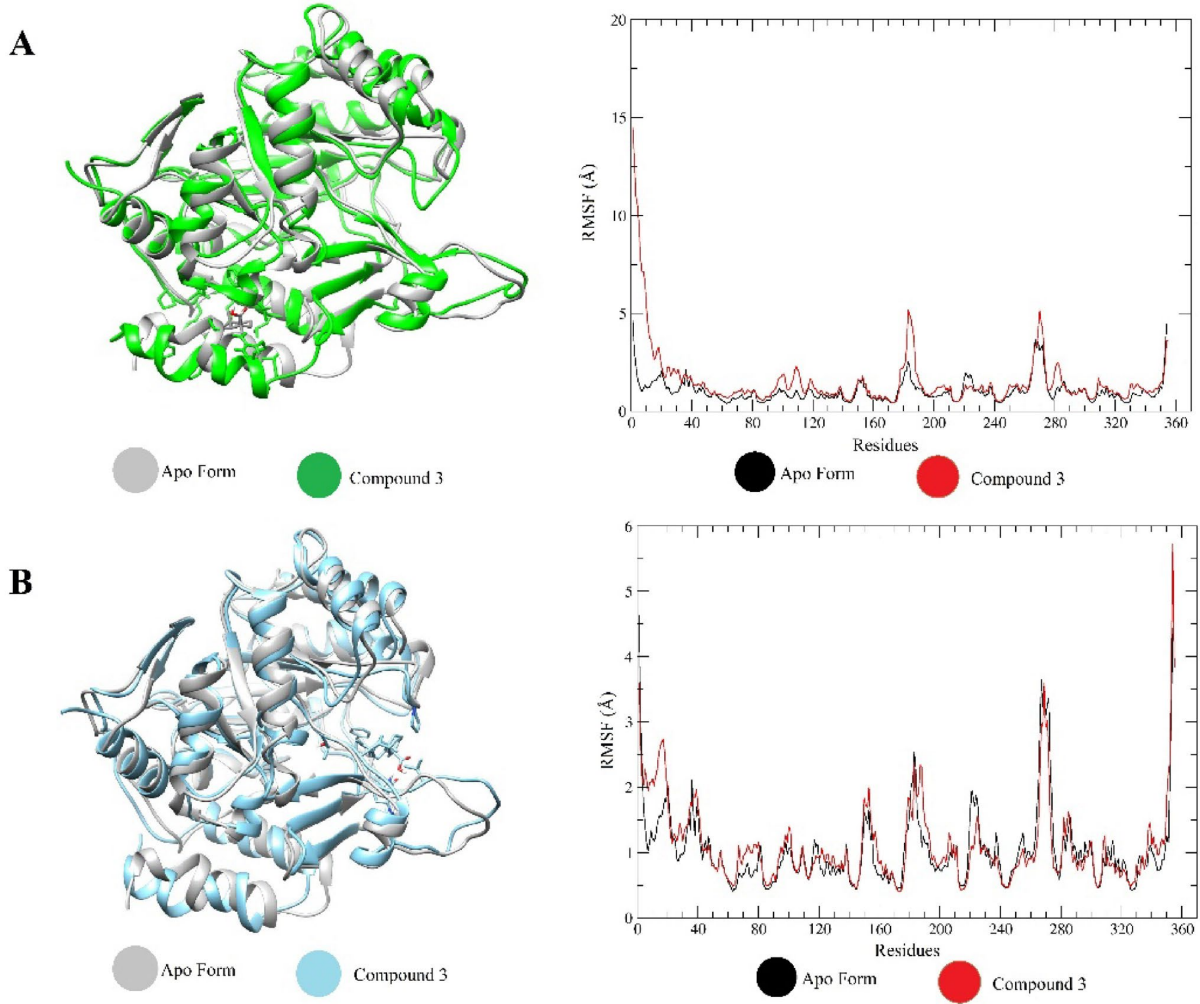
RMSF plots for the most stable structures of compound 3 at the binding sites 1 (**A**) and 2 (**B**) of the *Sm*DHODH enzyme

On the other hand, the occupancy of site 2 by compound 3 resulted in more discrete conformational oscillations from the RMSD and RMSF analyses, suggesting a lesser alteration of the enzyme’s global structure. Figure 11 displays the RMSF values for complex 3 (compound 3 at both binding sites under analysis). The results for the other complexes are provided in Supporting Information (Fig. S7 a, b, c and d).

The structural integrity and compactness of the *Sm*DHODH complexes were also monitored throughout the 120 ns of production. The stability observed in the RMSD profiles, with values for the protein backbone consistently remaining below 2.5 Å, indicates that the global enzyme conformation maintained its native compactness during the ligand-protein interactions. This structural consistency, along with the RMSF analysis that showed fluctuations restricted to specific loop regions, confirms that the catalytic domain remained well-packed and stable, without significant expansion or loss of folding throughout the simulation period.

**Fig. 12:**
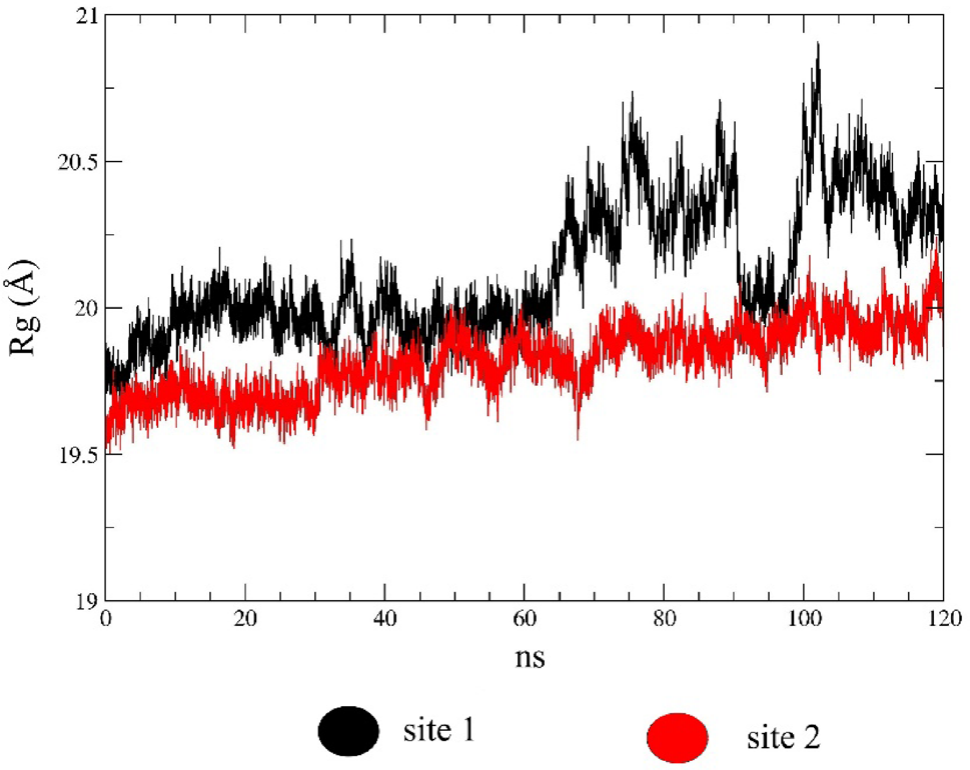
Sites

RMSD and RMSF analyses showed significant structural variations in the studied complexes, particularly in the complex containing compound 3 bound to site 1. Residues, in particular the region near site 2 with the set of loops, exhibited more significant fluctuations, indicating a large conformational change in this region. To complement the structural analysis of the complex formed by the target and compound 3 interacting at both sites, the radius of gyration (Rg) analysis was performed and the results are displayed in Fig. 12.

In MD simulations, the radius of gyration (Rg) provides insight into the structural compactness of the protein. It is calculated as the root means square distance of all protein atoms relative to the center of mass. Analysis of *Sm*DHODH bound to compound 3 at two sites revealed that the ligand binding at site 1 induces greater flexibility of the protein structure, potentially impacting the binding affinity. On the other hand, at site 2, the Rg variation is lower, indicating a more compact and less flexible region of the protein when compound 3 is bound to site 1. For a more accurate assessment of the complex stability, we estimated the values of free binding energy using the MM-GBSA (Molecular Mechanics-Generalized Born Surface Area) and MM-PBSA (Molecular Mechanics-Poisson-Boltzmann Surface Area) methods.

### Calculations of free binding energy

In addition to analyzing the RMSD and RMSF results, we performed calculations of the free binding energy between the five studied compounds (including PZQ) and the evaluated binding sites of the target to find correlations between the biological activity and the energy of the ligand-receptor complexes. To achieve this, implicit solvation methods (MM-GBSA and MM-PBSA) were employed. From Table 5, we verified that compounds 3 and 4 had the lowest values of free binding energy, suggesting greater stability in the interactions at both sites. The values obtained for site 1 indicated a higher binding affinity of the compounds, which may be caused by a network of hydrophobic contacts, primarily occurring at this site, potentially reducing the interaction energy significantly. These analyses corroborate the results obtained from the molecular docking simulations and the RMSD values.

**Table 5:** Binding free energy values (ΔG, kcal/mol) for the complexes formed between the selected molecules and *Sm*DHODH at binding sites 1 and 2, along with biological activity data obtained from previous studies (Sessa et al. 2020; Brito et al. 2022)

Compound	ΔG | MM-GBSA	ΔG | MM-PBSA	Biological activity (µM)
*Site 1*			
1	− 30.53	− 27.74	EC_50_ = 22.9
2	− 35.11	− 30.34	EC_50_ = 12.6
3	− 37.95	− 39.06	LC_50_ = 26.1
4	− 70.77	− 75.11	LC_50_ = 11.6
PZQ	− 45.55	− 40.41	LC_50_ = 1.1
*Site 2*			
1	− 28.37	− 22.38	EC_50_ = 22.9
2	− 23.88	− 15.93	EC_50_ = 12.6
3	− 33.71	− 39.42	LC_50_ = 26.1
4	− 53.62	− 60.90	LC_50_ = 11.6
PZQ	− 30.58	− 26.85	LC_50_ = 1.1

### Inhibition assays of enzymatic activity

Based on the results obtained from molecular docking studies, MD simulations, and calculations of free binding energy, compounds 3 and 4 were selected for inhibition assays of enzymatic activity (de Mori et al. 2021). The percentage of *Sm*DHODH activity for each compound is presented in Table 6.

**Table 6:** Residual enzymatic activity percentage of *Sm*DHODH after incubation with compounds 3 and 4 at 250 µM for 0 and 2 h

Compound (250 µM)	Activity *Sm*DHODH (%)	
	0 h	2 h
3	76 ± 2	40 ± 2
4	97 ± 4	90 ± 1

Activity was measured using a continuous colorimetric assay, with DCIP reduction as the indicator. The data represent the mean of three independent experiments ± standard deviation

From Table 6, we observed that compound 3 exhibited superior performance in inhibiting the *Sm*DHODH enzyme, confirming that hydrophobic interactions play a key role in the stability of the complex, particularly when considering the entrance regions of sites 1 and 2. This result is consistent with previous reports of other *Sm*DHODH inhibitors that also act in the micromolar range (e.g., chloroquine, primaquine, quinine, and mefloquine), indicating that even moderate enzyme inhibition can be relevant in the search for new lead compounds, particularly when safety and accessibility are considered essential factors for neglected diseases. A comparative table with previously reported *Sm*DHODH inhibitors, along with their potency, and toxicity profiles is provided in Supplementary Information (Table S8).

**Fig. 13:**
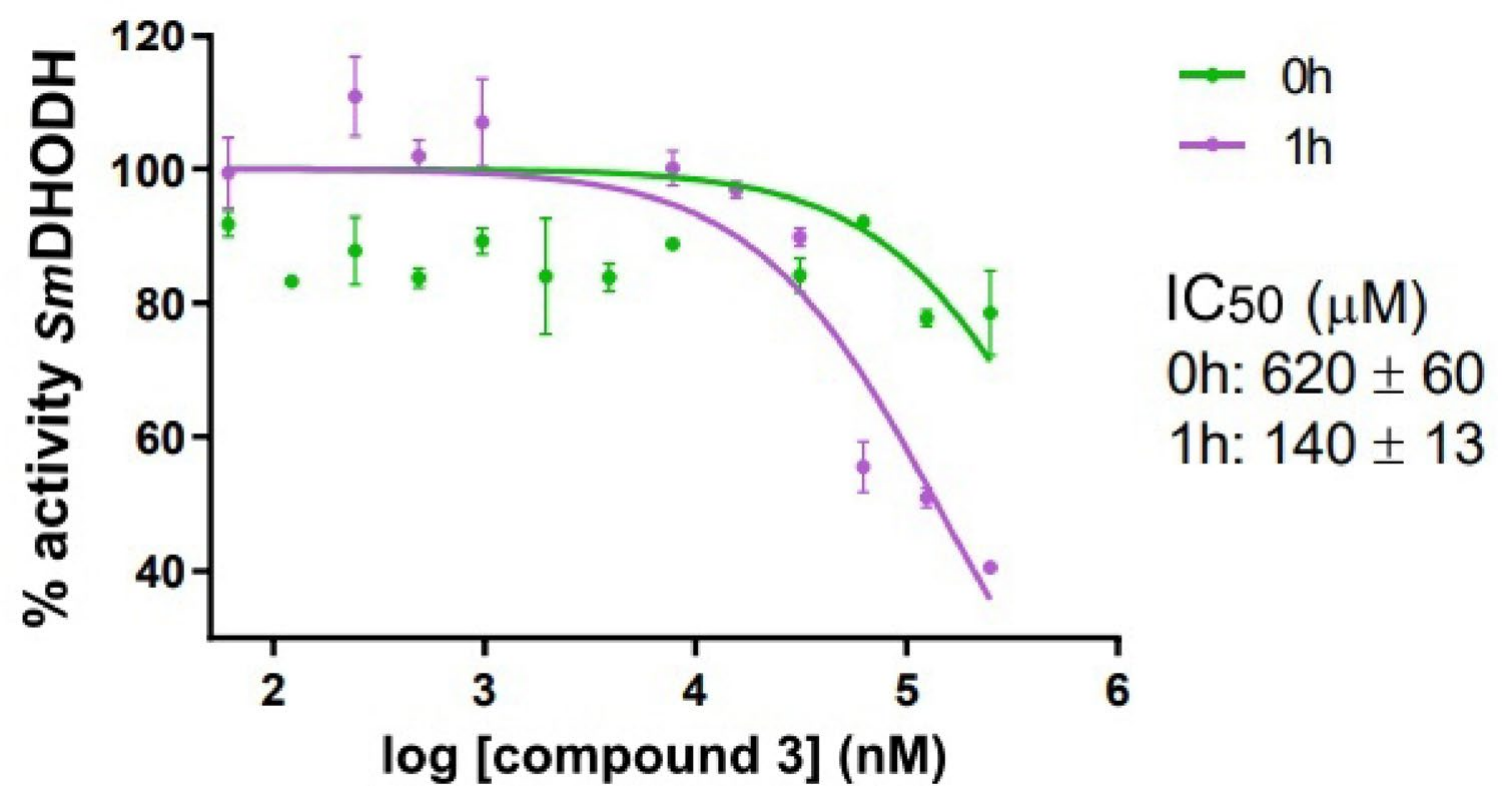
Inhibition curves of *Sm*DHODH for compound 3 after incubation (0 and 2 h). The enzyme was incubated with different concentrations of compound 3, and the residual activity was measured

Comparing the values of log P and TPSA for the compounds under analysis and the hydrophobicity characteristics of the binding sites (Table S9 and Fig. S10), we identified structural differences that may influence cellular permeation and the bioavailability of the substances. Although compound 4 has a lower log P, its predominantly polar nature may have hindered its effective insertion into the enzyme’s active site, as the binding site entrance has more hydrophobic profile, which would impair the formation of a stable complex. Figure 13 displays the inhibition curves of *Sm*DHODH and the IC_50_ values for compound 3.

### Suggestions of structural optimization for compound 3 (ent-kaur-16-en-19-oic acid)

**Fig. 14:**
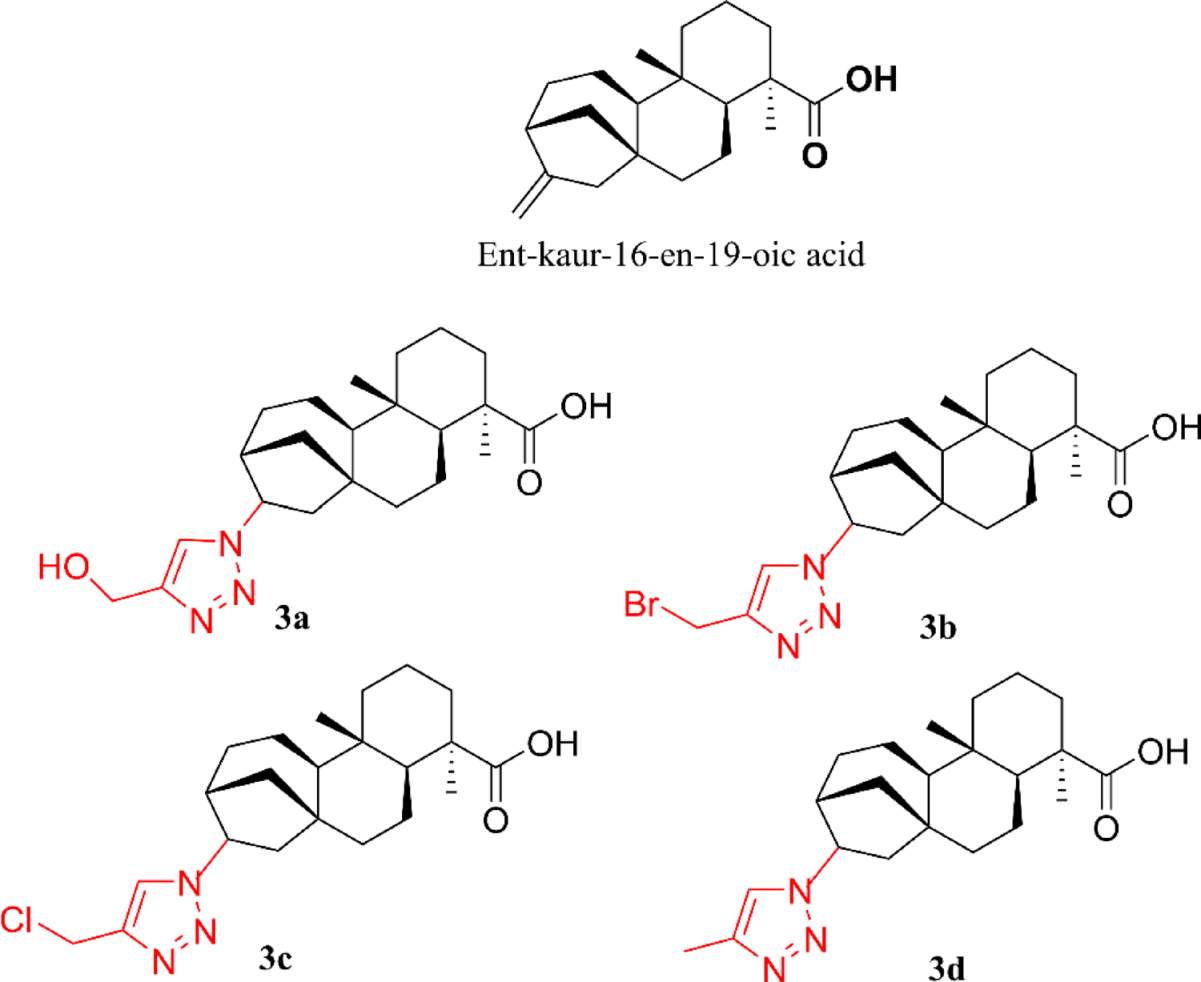
Structural modifications of *ent*-kaur-16-en-19-oic acid (compound 3). The starting compound (top) was modified by replacing the exocyclic double bond (Δ16) with a triazole ring (highlighted in red) and different substituents (3a: hydroxymethyl; 3b: bromine; 3c: chloromethyl; 3d: dimethyl). The diterpenoid skeleton and the carboxylic group (C-19) were preserved

**Fig. 15:**
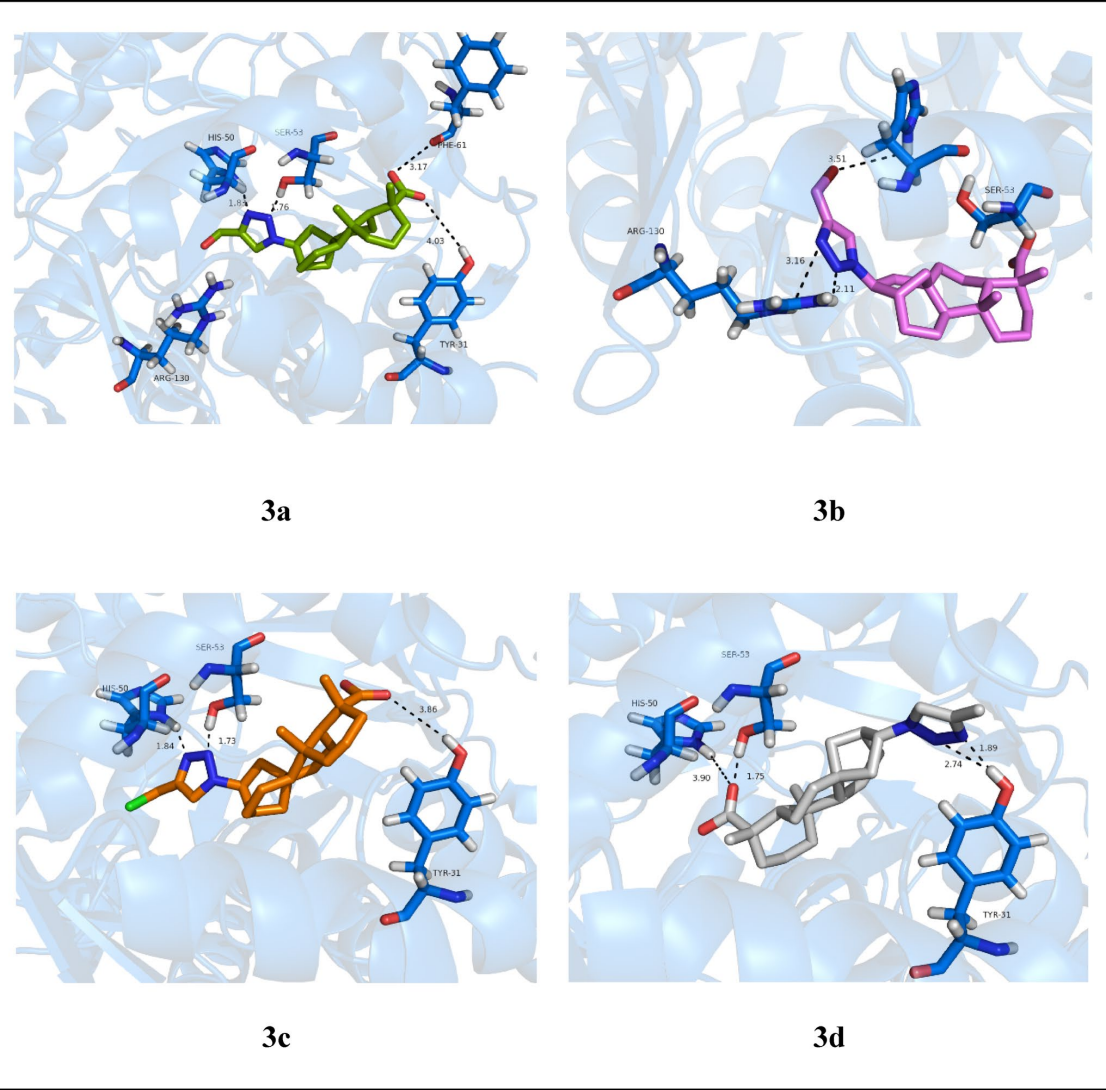
Molecular docking results of derivatives 3a, 3b, 3c, and 3d, highlighting hydrogen-bonding interactions with key residues (His50,Ser53, Tyr31, and Arg130) at site 1 of *Sm*DHODH

Based on the orientation of key residues in site 1, such as Tyr31 and Arg130, which are crucial for the ligand stabilization but did not interact with the original compound (3), an in silico strategy of molecular optimization was designed. This strategy was guided by the results obtained from the binding site characterization, docking, and molecular dynamics studies. The introduction of a triazole ring at the C-16/C-17 position of compound 3 yielded derivatives 3a–3d (Fig. 14). Figure 15 shows the best-energy conformations of the new derivatives from docking studies at site 1 of the *Sm*DHODH enzyme.

The docking results for the derivatives of compound 3 (Table 7) showed the molecular modifications led to a significant improvement in the estimated binding free energy (ΔG). We can also see that the derivatives 3a, 3c, and 3d performed hydrogen-bonding interactions with Tyr31 without altering the hydrophobic regions.

**Table 7:** Key results from the docking studies of derivatives 3a to 3d at site 1 of *Sm*DHODH

Compound	Binding Interactions		No of interactions (H-bond)	H-bonds	Hydrophobic interactions
	ΔG (kcal/mol)	Fitness			
3a	− 38.11	35.35	4	His50, Ser53, Phe61, Tyr31	Tyr31, Phe35, Leu36, Leu56, Phe357, Pro362
3b	− 31.42	30.32	3	His50, Arg130	Phe35, Leu36, Ala39, Ala49, Phe357, Val358
3c	− 40.95	36.50	3	His50, Ser53, Tyr31	Phe35, Leu36, Leu56, Phe357, Val358, Pro362
3d	− 40.59	36.49	3	Tyr31, His50 Ser53	Tyr31, Leu36, Leu56, Ile62, Phe357, Pro362

The values of ΔG and fitness estimated from ChemScore (scoring function) indicate the potential binding affinity

The results indicated that the structural and polar optimization of compound 3 can increase the affinity and, potentially, the inhibitory capacity. Therefore, this study offers a rational way to design natural product-derived analogues with greater specificity and potency related to anti-schistosomal activity.

### Limitations and future perspectives

Despite the promising inhibition observed for compound **3**, some limitations should be noted. The high lipophilicity of this diterpene, as indicated by its log P value (Table S9), may present challenges for aqueous solubility and effective penetration through the parasite’s lipid-rich tegument. To address these challenges, future research will focus on the synthesis and biological evaluation of the triazole derivatives (3a–3d) proposed in this study, aiming to improve polar interactions and optimize the pharmacokinetic profile of the kaurane scaffold against *Sm*DHODH.

## Conclusions

This study presents the *Sm*DHODH enzyme as a promising target for treating schistosomiasis and suggests a new potential binding site. Natural products 3 (*ent*-kaur-16-en-19-oic acid) and 4 (15β-senecioil-oxi-*ent*-kaur-16-en-19-oic acid) showed the best binding energy values, indicating greater stability at site 1 of the biological target. Molecular dynamics analyses indicated that compound 3 induced significant conformational changes in the *Sm*DHODH structure, especially in the loop regions near the site 2, suggesting the enzyme’s structural flexibility upon binding with the ligand. However, the biological assays indicated that compound 3 might require incubation time to properly penetrate the active site or induce the structural rearrangements necessary for the enzyme inhibition. These findings are consistent with the experimental data, which suggest an increase in inhibitory efficacy after 1 h of incubation. Two key challenges emerge from these results: (1) improving solubility, since both binding sites contain many apolar residues, and (2) ensuring effective penetration of compounds through the parasite’s thick, lipid-rich tegument. Building upon the computational findings and experimental enzymatic results, structural modifications were subsequently suggested on compound 3. These changes were guided by its optimal binding conformation within the pocket to enhance interactions with key residues and increase its inhibitory potential. From our strategy for molecular modification targeting *Sm*DHODH, four novel derivatives of *ent*-kaur-16-en-19-oic acid (compound 3) were proposed for future synthesis and in vitro evaluation.

## Supplementary Information

Below is the link to the electronic supplementary material.

Supplementary Material 1

## Data Availability

Upon reasonable request, the corresponding author will provide the information supporting the study’s findings.
