# Supplementary material for: Exploring SmDHODH inhibition: natural products with potential anti-schistosomiasis activity
Source: In Silico Pharmacol. 2026 May 8;14(2):137. doi: 10.1007/s40203-026-00628-1 (PMC13156374; doi:10.1007/s40203-026-00628-1)
Supplement: Supplementary file 1 — Supplementary Material 1 [file 40203_2026_628_MOESM1_ESM.docx]

**Supplementary Information**

**Exploring *Sm*DHODH Inhibition: Natural Products with Potential Anti-Schistosomiasis Activity**

Rafaela Molina de Angelo^1^, Michell de Oliveira Almeida^2^, João Pedro Portilho Encide^1^, Henrique Barbosa^1^, Daniel da Silva de Sousa^3^, Aldineia Pereira da Silva^4^, Marina Sena Mendes^5^, Maria Cristina Nonato^5^, Albérico Borges Ferreira da Silva^3^, João Henrique Ghilardi Lago^1*^, Kathia Maria Honorio^1,4*^

*^1^Center of Natural and Human Sciences (CCNH) - Federal University of ABC (UFABC), Santo André, SP, Brazil.*

*^2^Faculty of Pharmaceutical Sciences (FCF) - University of São Paulo (USP), São Paulo, SP, Brazil.*

*^3^São Carlos Institute of Chemistry (IQSC) – University of São Paulo (USP), São Carlos, SP, Brazil.*

*^4^School of Arts, Sciences and Humanities (EACH) - University of São Paulo (USP), São Paulo, SP, Brazil*

*^5^School of Pharmaceutical Sciences at Ribeirao Preto (FCFRP), University of São Paulo (USP), Ribeirão Preto, SP, Brazil.*

*Corresponding authors (e-mail: kmhonorio@usp.br and joao.lago@ufabc.edu.br)

**Table S1**

Table S1 is organized in descending order of the biological activity values, from the highest to lowest activity compound, followed by praziquantel (PZQ) as a positive control. It is important to note that compounds **3** and **4** were experimentally tested to determine the lethal concentration at 50% (LC_50_), while compounds **1** and **2** were assessed for their effective concentration at 50% (EC_50_). All compounds were evaluated for selectivity and tested on HaCat mammalian cells to assess cytotoxicity.

**Table S1**. Biological activity, selectivity, and cytotoxicity values against *S. mansoni* for the studied compounds **1 - 4**, including PZQ. The values highlighted in blue were used to select the best compounds based on each obtained property

| **Compound** | **LC_50_ (μM)** | **EC_50_ (μM)** | **CC_50_ (μM)** | **SI** |
| --- | --- | --- | --- | --- |
| **1** | - | 22.9 (18.2-28.6) | >500 | > 17.8 |
| **2** | - | 12.6 (10.8-18.3) | >500 | > 26.8 |
| **3** | 26.1 (23.7-32.4) | - | 108.6 (93.2-138.4) | 4.2 |
| **4** | 11.6 (8.4-15.1) | - | 147.2 (118.6-170.8) | 12.7 |
| **PZQ** | 1.1 (0.4-1.8) | 0.7 (0.6-0.8) | >200 | >180 |

LC_50_: lethal concentration 50% against adult schistosomes; EC_50_: effective concentration 50% against adult schistosomes; CC_50_: cytotoxic concentration 50% against mammalian cells HaCat human keratinocyte. SI: selectivity index.

**Figure S2 - Protein Preparation and Binding Site**

Results from the predictions performed by the SwissTargetPrediction server were used to identify receptor classes with potential interactions with the natural products studied (see Figure S2).

**
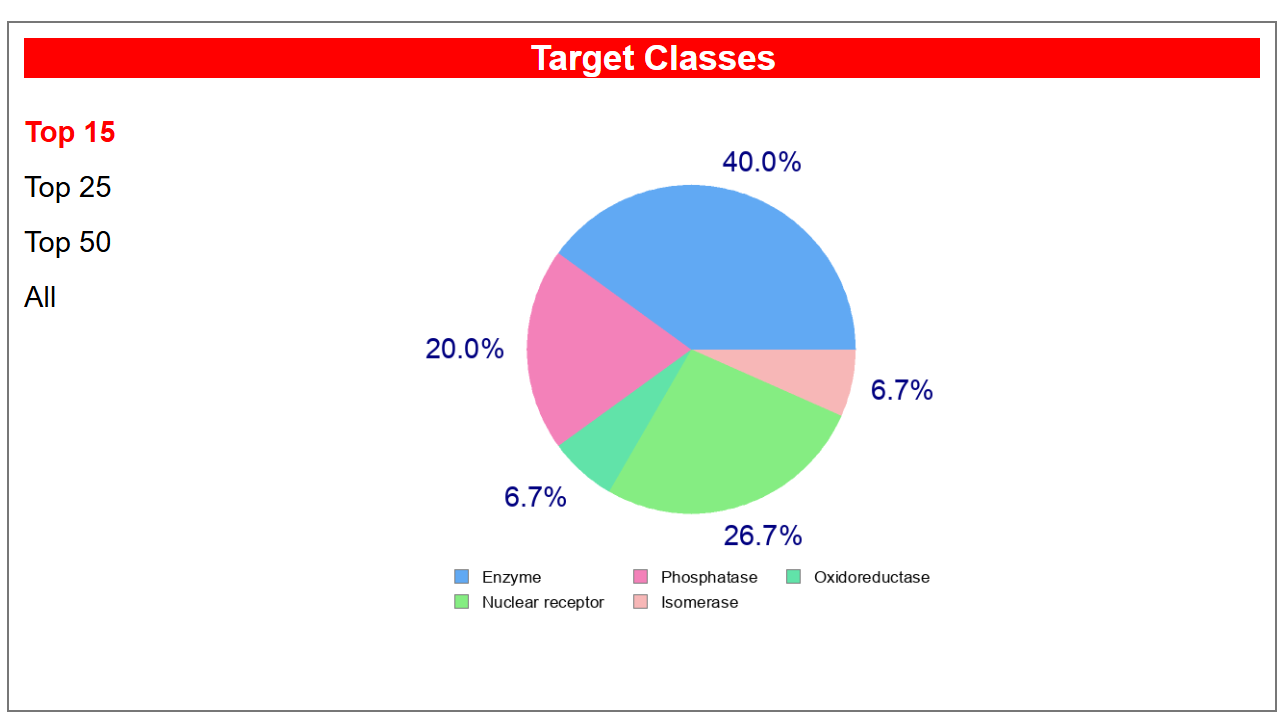
**

**
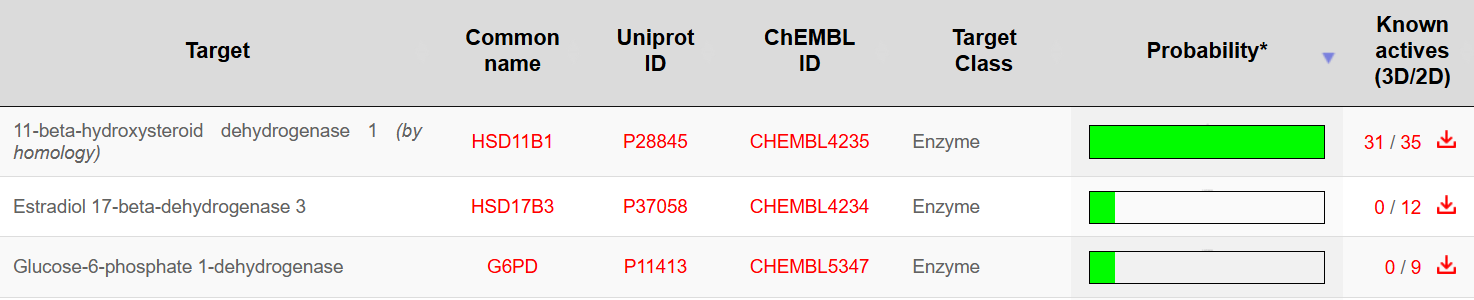
**

**Figure S2.** Predicted targets for the studied natural products, highlighting the highest probability match with 11β-hydroxysteroid dehydrogenase type 1 (HSD11B1). Information includes common names, UniProt IDs, ChEMBL IDs, target class (enzyme), prediction probability, and number of known active compounds (3D/2D similarity). Pie chart showing the distribution of the top 15 predicted targets based on target classes. The majority belong to enzymes (40%), followed by nuclear receptors (26.7%), phosphatases (20%), isomerases (6.7%), and oxidoreductases (6.7%).

The 11β-hydroxysteroid dehydrogenase type 1 (11β-HSD1) shares structural similarities with *Sm*DHODH (dihydroorotate dehydrogenase from *Schistosoma mansoni*), particularly regarding cofactor. Both enzymes belong to the same class of dehydrogenases, which are involved in oxidation-reduction reactions and utilize cofactors such as NAD(P)+/NAD(P)H. The similarity is especially evident in their amino acid sequences, which can be used to study homology between these enzymes and explore potential therapeutic targets.

**Figure S3 – Characterization of binding sites**


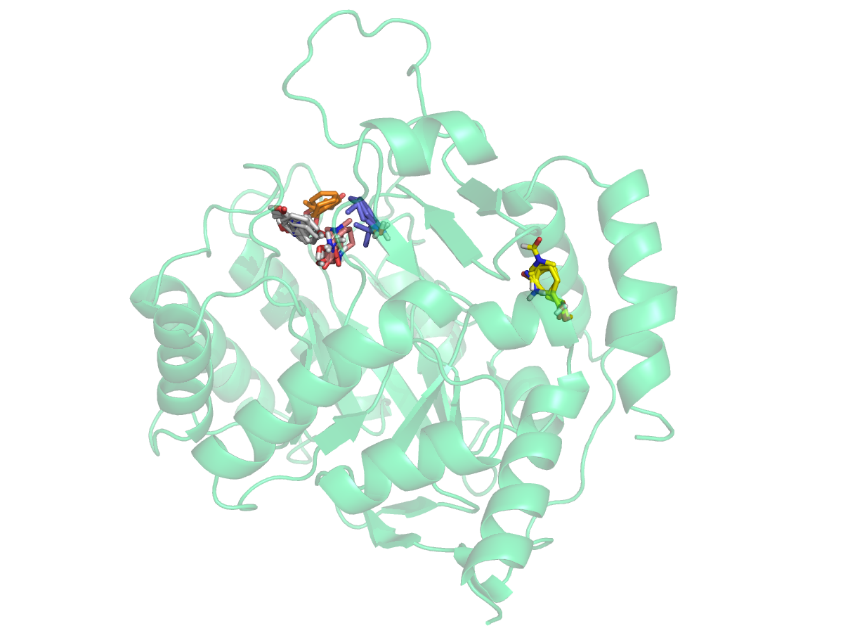


**Figure S3.** Probes found by the FTmap server, which were used to characterize the binding sites and to understand the possible interactions in each of them. The probe in yellow is placed at site 1, while the set of probes on the left is located within the pocket of site 2 (potential allosteric site).

**Structural Analyses of Binding Sites (S4)**

**Table S4.** Classification of amino acid residues in the interconnected binding pockets

| **Residue Category** | **Amino Acid Residues and Positions** |
| --- | --- |
| Acidic (Negative) | Asp45, Asp76, Asp277, Asp318, Glu47, Glu111, Glu123, Glu135, Glu204, Glu275 |
| Basic (Positive) | Arg125, Arg149, Arg173, Arg210, Arg305, Arg307, Arg308, Arg310, Arg361 |
| Polar (Uncharged) | Asn51, Asn123, Asn161, Asn191, Asn197, Asn292, Asn306, Gln57, Gln111, Gln352, Gln360, Ser35, Ser42, Ser98, Ser127, Ser137, Ser176, Ser271, Thr190, Cys141 |
| Non-Polar (Aliphatic) | Ala48, Ala56, Ala97, Ala98, Ala107, Ala127, Ala134, Ala136, Ala140, Ala142, Ala143, Ala161, Ala177, Ala199, Ala206, Ala211, Ala274, Ala290, Ala301, Ala307, Ala309, Gly110, Gly122, Gly124, Gly135, Gly310, Gly361, Leu41, Leu135, Leu204, Leu246, Leu306, Pro110, Pro126, Pro135, Pro244, Pro247, Pro290, Pro307, Pro363 |
| Aromatic | Phe41, Phe61, Phe92, Phe95, Phe97, Phe357, Tyr31, Tyr41, Tyr53, Tyr173, Tyr183, Tyr314, Tyr354, Tyr357; |

**Figure S5 - Analyses of Movement and Flexibility of the Target Structure**

Figure S5(a) shows the eigenvalues associated with each mode of the enzyme movement, with the mode index ranging from 1 to 20 on the x-axis and the eigenvalues in kJ/mol on the y-axis. The eigenvalues represent the rigidity of the movement. The higher the eigenvalue, the more energy is required to deform the enzyme structure. This result indicates that modes with higher eigenvalues are more rigid and less prone to deformation. Figure S5(b) illustrates the variance associated with each mode of movement, with the mode index ranging from 1 to 20 on the x-axis and the variance as a percentage on the y-axis. The red bars represent individual variances, while the green bars show cumulative variances. Variance indicates how much each mode contributes to the overall movement of the enzyme. Modes with higher variance have a more significant impact on the overall movement. Variance is inversely related to eigenvalues, meaning that modes with lower eigenvalues have higher variance and contribute more to the flexibility of the structure. These results are essential for understanding how different modes of movement affect the structure of the *Sm*DHODH enzyme, which may be crucial for studying its function and interactions with other molecules.

| 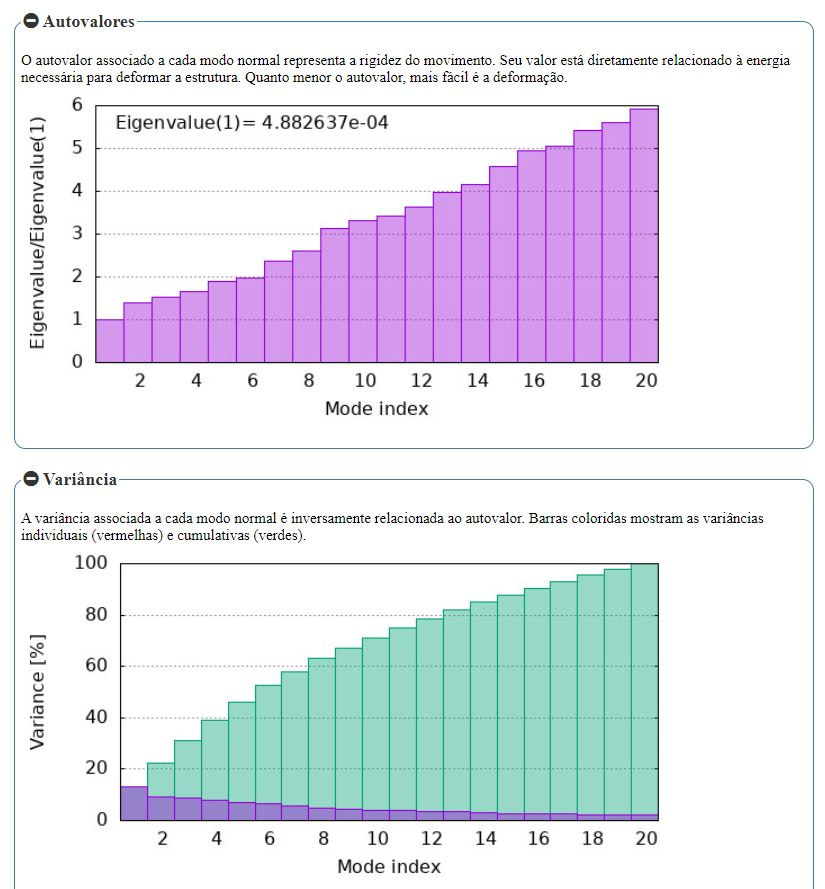 | 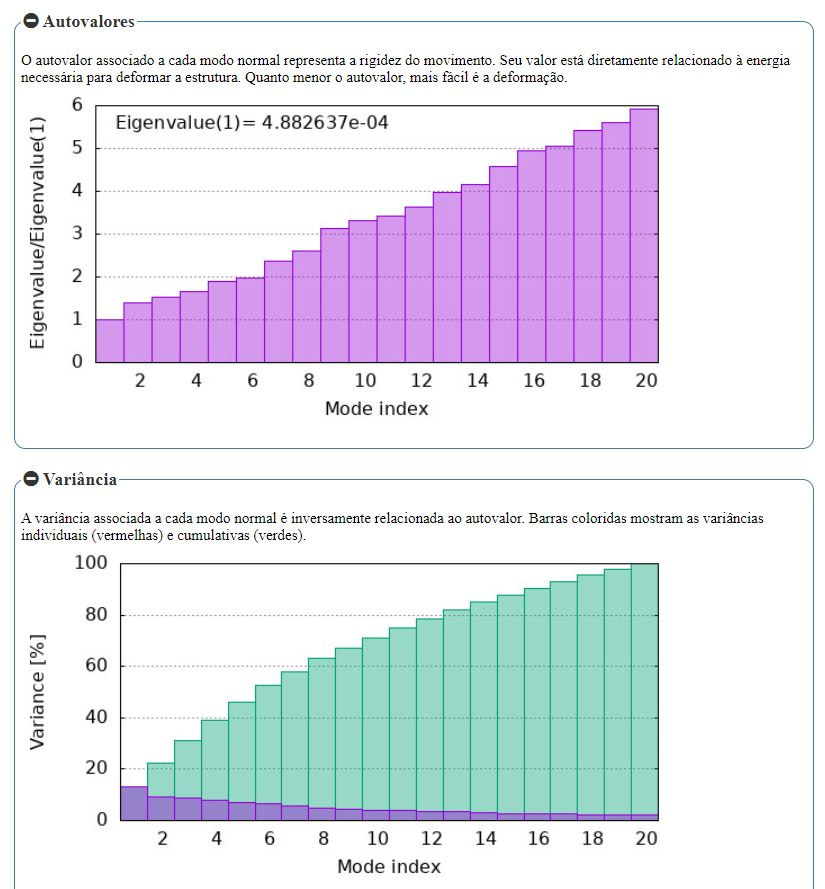 |
| --- | --- |

1. **(b)**

**Figure S5. (a)** The eigenvalue associated with each normal mode represents the rigidity of the movement. Its value is directly related to the energy required to deform the structure; **(b)** the variance associated with each normal mode is inversely related to the eigenvalue — colored bars: individual (red) and cumulative variances (green).

**Figure S6 - Molecular Dynamics (MD) Simulations**

The initial MD analysis involved evaluating the RMSD values. Figures S6 (a), (b), (c) and (d) display the RMSD values of the complexes formed by the remaining compounds at both binding sites (sites 1 and 2) of the *Sm*DHODH enzyme.


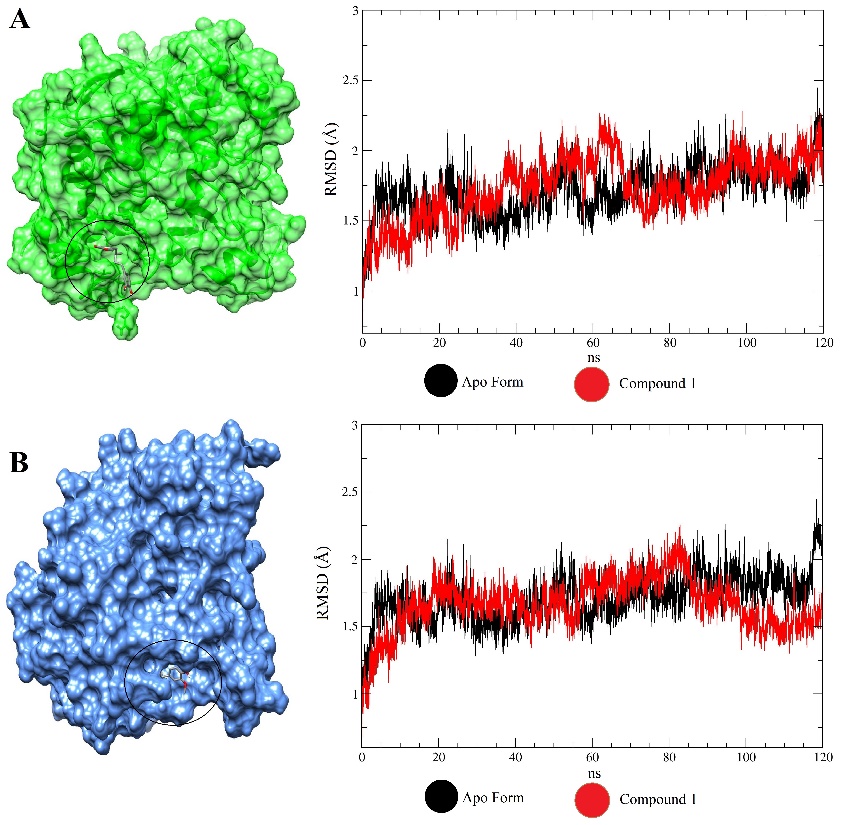


**Figure S6 (a).** RMSD results for (A) compound **1** at site 1 (green-colored protein); (B) compound **1** at site 2 (blue).


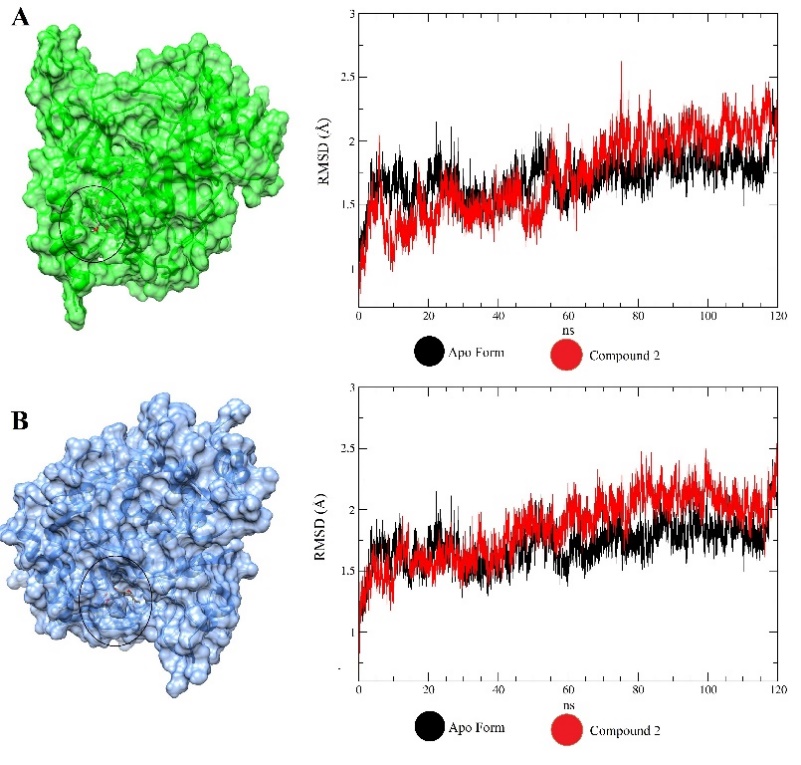


**Figure S6 (b)**. RMSD results for (A) compound **2** at site 1 (green-colored protein); (B) compound **2** at site 2 (blue).


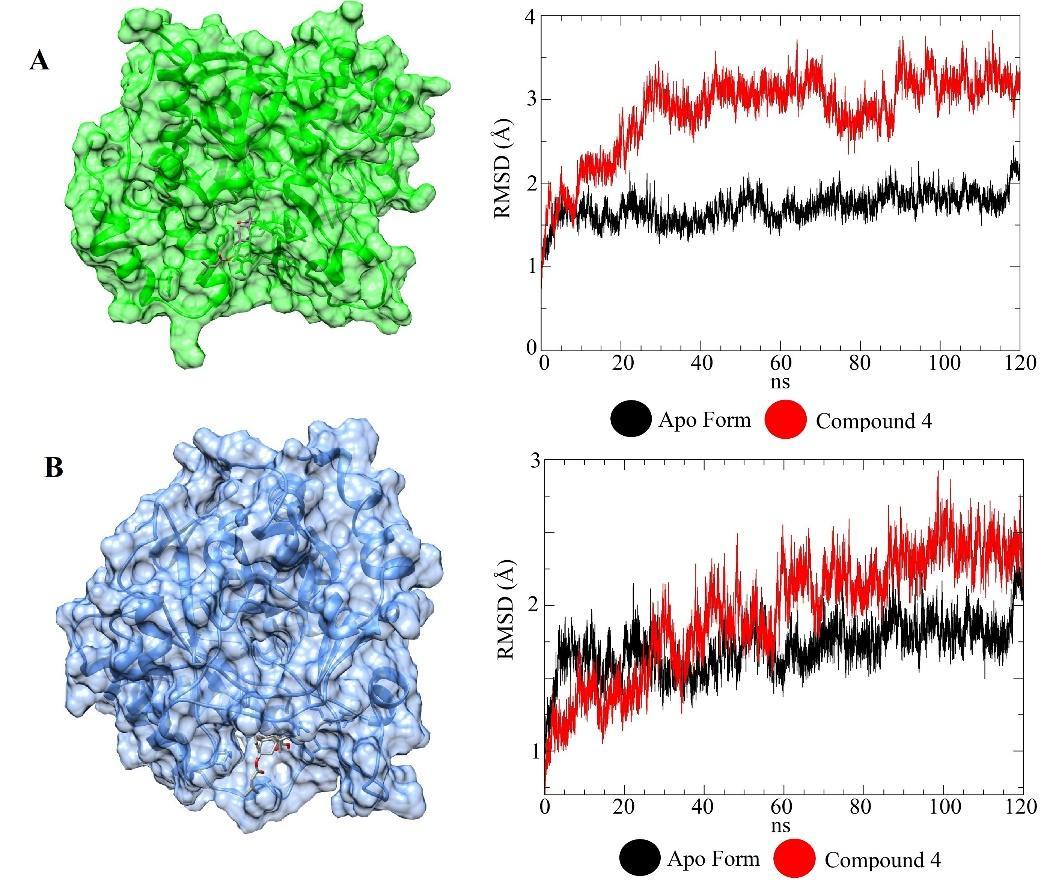


**Figure S6 (c)**. RMSD results for (A) compound **4** at site 1 (green-colored protein); (B) compound **4** at site 2 (blue).


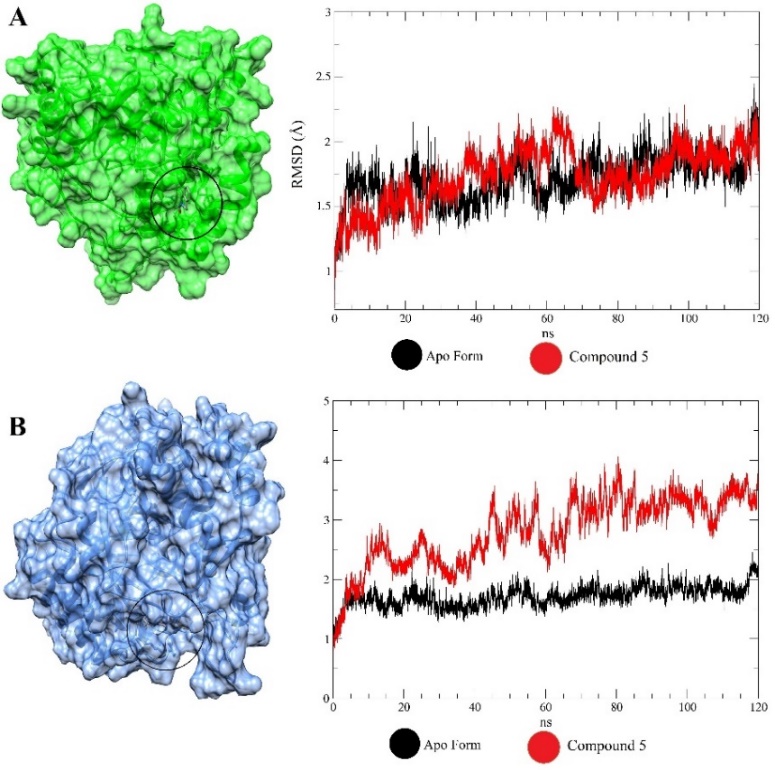


**Figure S6 (d).** RMSD results for (A) compound **5** at site 1 (green-colored protein); (B) compound **5** at site 2 (blue).

**Figure S7 – RMSF analyses**

RMSF values were also analyzed, indicating the levels of fluctuation of amino acid residues in the *Sm*DHODH binding sites, considering the presence of the other four studied compounds. Figures S7 (a), (b), (c) and (d) present the RMSF results.


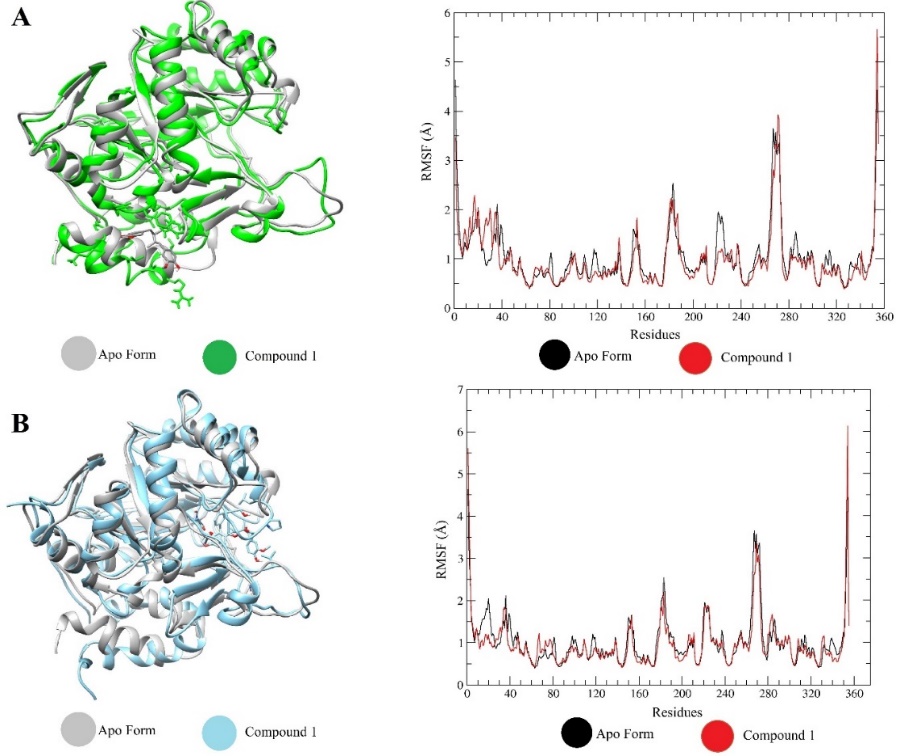


**Figure S7 (a).** RMSF plots used to analyze the fluctuation levels of amino acid residues with compound **1** at sites 1 (A) and 2 (B) of the *Sm*DHODH enzyme.


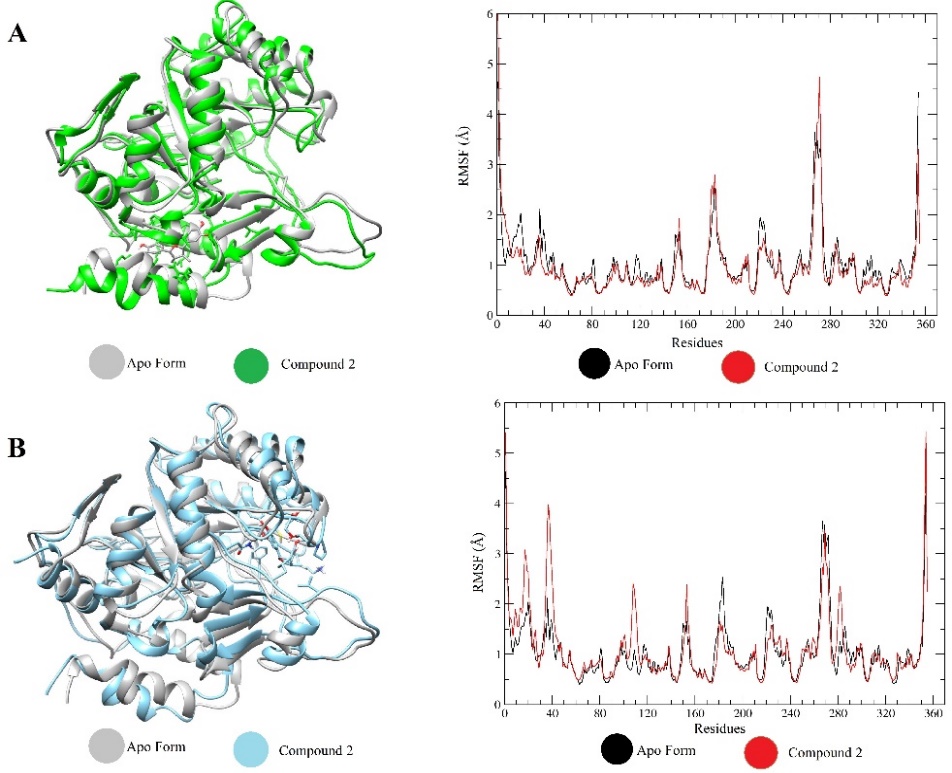


**Figure S7 (b).** RMSF plots used to analyze the fluctuation levels of amino acid residues with compound **2** at sites 1 (A) and 2 (B) of the *Sm*DHODH enzyme.


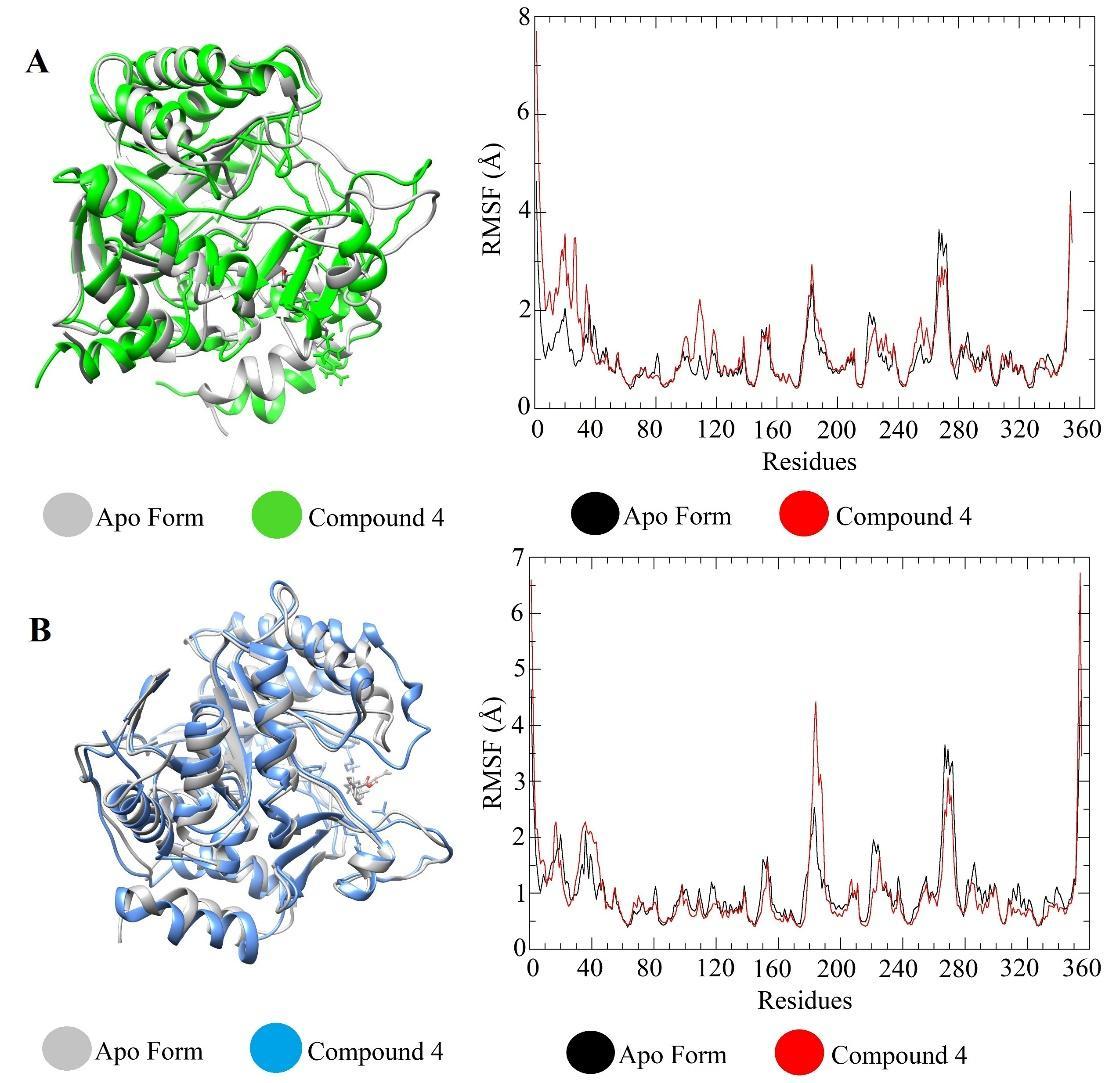


**Figure S7 (c).** RMSF plots used to analyze the fluctuation levels of amino acid residues with compound **4** at sites 1 (A) and 2 (B) of the *Sm*DHODH enzyme.


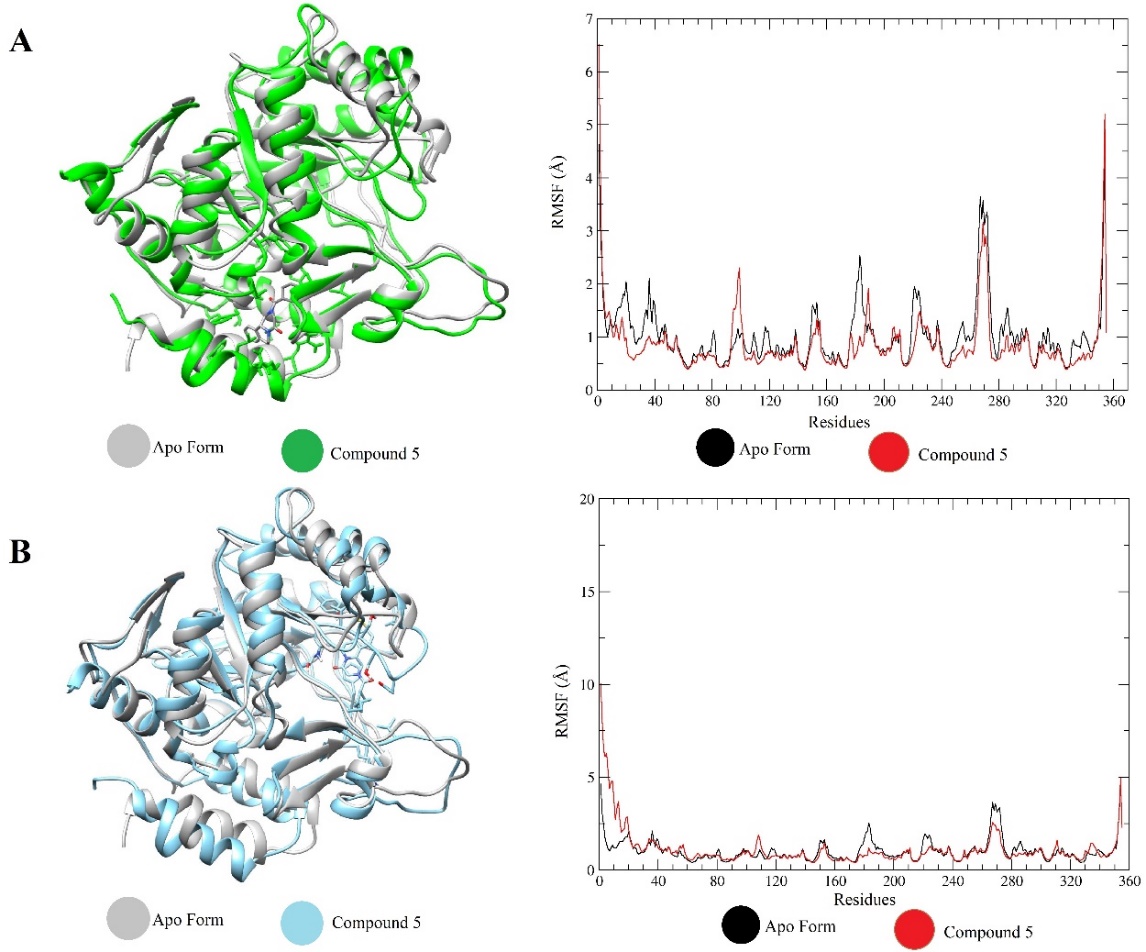


**Figure S7 (d).** RMSF plots used to analyze the fluctuation levels of amino acid residues with positive control drug praziquantel (PZQ - compound **5**) at sites 1 (A) and 2 (B) of the *Sm*DHODH enzyme.

**Table S8 - *Sm*DHODH inhibitors reported in the literature**

**Table S8.** Comparison of known *Sm*DHODH inhibitors, their potency (IC₅₀), *in vivo* and *in vitro* evaluation, and reported toxicity profiles. The table includes highly potent (nM) inhibitors and weaker (μM) compounds previously studied. This comparison highlights that natural products with lower human toxicity, such as compound **3** (identified in this study), are relevant as promising lead compounds for optimization in the context of a neglected disease

| **Compound** | **Origin** | **IC_50_ (*Sm*DHODH)** | ***in vivo / in vitro* studies** | **Toxicity /  *in vivo* data** | **Reference** |
| --- | --- | --- | --- | --- | --- |
| Lapachol (2-hydroxy-3-(3-methyl-2-butenyl)-1,4-naphthoquinone) | Natural (*Tabebuia* species) | ~100 nM | *in vivo* tested (mice; prophylaxis of schistosomiasis) | Reproductive and hemolytic toxicity in rodents (hemolytic anemia, embryo-toxicity); toxic at high systemic doses in rats, abortive effects; no human schistosomiasis data | Lima *et al*., 2002; PubMed 12062792 |
| Atovaquone (antimalarial, naphthoquinone derivative) | Synthetic | ~432 nM | *in vitro* (adult *S. mansoni* worms; schistosomicidal activity observed) | Low to moderate clinical toxicity; rare hepatotoxicity; non-teratogenic in rats; no specific *in vivo* tests for *Sm*DHODH | LiverTox (NCBI); FDA label |
| QLA – 2-((4-fluorophenyl)amino)-3-hydroxy-1,4-naphthoquinone (atovaquone analogue) | Synthetic | ~227 nM | – | No published clinical or *in vivo* toxicity data; expected profile similar to atovaquone but lacking preclinical toxicology studies | Calil *et al*., 2019; de Mori *et al*., 2021 |
| Compound 17 – 2-hydroxy-3-isopentylnaphthalene-1,4-dione (semisynthetic lapachol derivative) | Semisynthetic (derived from lapachol) | 23 nM | – | Potential embryo-toxicity inferred from lapachol structure; no specific *in vivo* data published for schistosomiasis | Calil *et al*., 2019 |
| Chloroquine | Synthetic | >250 µM | No significant inhibitory effect up to 250 µM; not used clinically for schistosomiasis | Well-known toxicity: retinal toxicity (irreversible with prolonged use), QT prolongation, arrhythmias; neurological and hepatic adverse effects | WHO malaria treatment manual; BMC Medicine 2018 |
| Primaquine | Synthetic | >250 µM | No inhibition up to 250 µM; ineffective against adult worms | Hemolytic risk in G6PD deficiency; methemoglobinemia; gastrointestinal intolerance (nausea, cramps) | CDC Malaria Factsheet; Clinical Infectious Diseases 2004 |
| Quinine | Natural (alkaloid from *Cinchona*) | >250 µM | No inhibition up to 250 µM | Cinchonism (tinnitus, blurred vision, headache, dizziness); high doses: arrhythmias, hemolysis (in G6PD), rare immune reactions | WHO malaria manual; Cinchonism review |
| Mefloquine | Synthetic | >250 µM | Negligible inhibition up to 250 µM; off-target effects in animal models | Persistent neurotoxicity (hallucinations, depression, seizures); contraindicated in psychiatric or seizure disorders; mild cardiotoxicity | BMC Public Health 2002; FDA drug safety |

**Table S9 - Lipinski’s Rules**

From the analysis of the estimated parameters and according to Lipinski's Rule of Five, we can state that a molecule, even if it violates one of the parameters, may still be a candidate as an orally bioavailable drug. Therefore, these rules provide a good prediction of oral bioavailability for new bioactive molecules. Analyzing the properties estimated in this study, it is interesting to note that the positive control (PZQ) does not violate Lipinski's rules, and its
log P value is significantly lower than the other compounds. Table S9 shows the values for each Lipinski property estimated for the studied compounds.

**Table S9.** Physicochemical properties used to predict the oral bioavailability of the studied compounds

| **Compound** | **Molecular mass (Da)** | **Num. H bond acceptors** | **Num. H bond donors** | **TPSA (Å)** | **Consensus Log *P*_o/w_** | **Lipinski violations** |
| --- | --- | --- | --- | --- | --- | --- |
| **1** | 328.40 | 4 | 1 | 47.92 | 4.13 | 0 |
| **2** | 344.40 | 5 | 2 | 68.15 | 3.06 | 0 |
| **3** | 302.45 | 2 | 1 | 37.30 | 4.44 | 1 violation: Log P >4.15 |
| **4** | 400.55 | 4 | 1 | 63.60 | 4.97 | 1 violation: Log P >4.15 |
| **PZQ** | 312.41 | 2 | 0 | 40.62 | 2.40 | 0 |

Total polar surface area (TPSA) refers to the total surface area of a molecule occupied by atoms with partial or complete charges. In other words, TPSA quantifies the polar portion of the molecule's surface and, consequently, is involved in intermolecular interactions such as hydrogen bonding, dipole-dipole interactions, and van der Waals forces. Compounds **2** and **4**, which exhibit the best biological activity values, have the highest TPSA values, indicating greater ease of interaction with the intermediate portion of the flexible loop at sites 2 and 1.

**Figure S10 – Suggestions of structural modifications**

The results suggest that the hydrophobicity of compound **3** is a determining factor for its ability to interact with the enzyme's active sites, especially in the hydrophobic regions at the entrance of the binding sites, which may have favored its greater inhibition of *Sm*DHODH compared to compound **4**. Figure S10 shows the hydrophobic interactions of compound **3** at sites 1 and 2 and a hydrophobicity surface map. The fit of compound **3** at site 1 is more favorable, as the binding pocket has apolar residues such as Pro362, Ile62, Pro63, and Phe357, which interact and stabilize the entire hydrophobic aromatic region, particularly the concentrated regions of the senecioyl moiety.


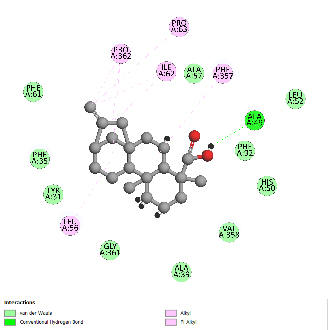

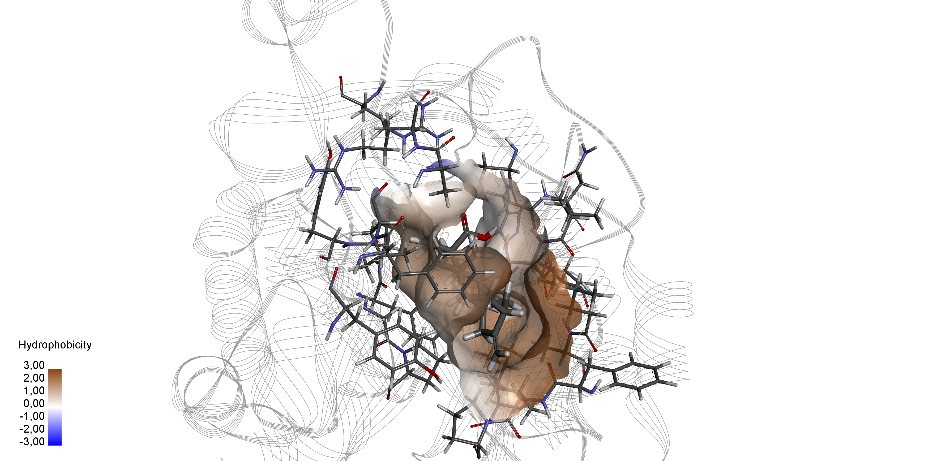


(a)


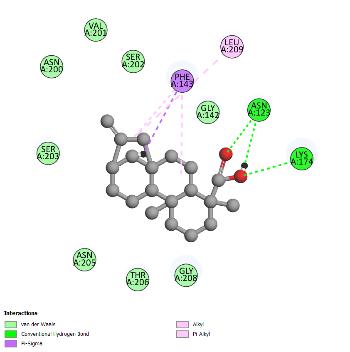

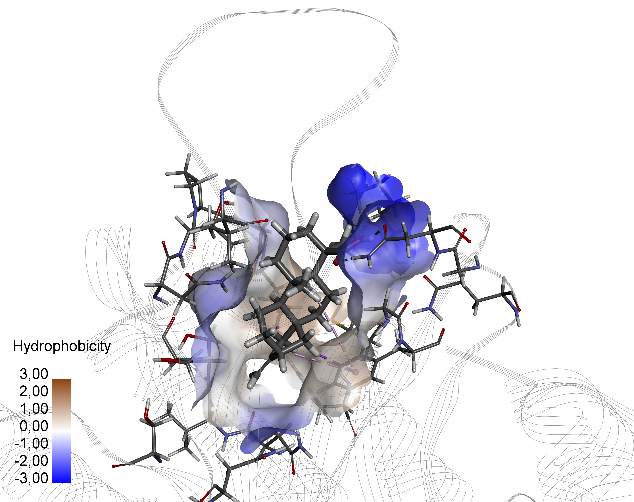


(b)

**Figure S10.** Hydrophobic interactions of compound **3** at sites 1 **(a)** and 2 **(b)** of *Sm*DHODH and a hydrophobicity surface map. Compound **3** exhibits hydrophobic regions, primarily the aromatic region of the naphthalene ring system and the concentrated regions of the senecioyl moiety, which contain methyl groups and other apolar characteristics that favor hydrophobic interactions. The orange surface highlights the hydrophobic regions, representing areas with higher apolar character that promote favorable interactions with other hydrophobic molecules.
